# Single-cell analysis reveals crosstalk between TREM1-positive myeloid cells and cancer-associated fibroblasts in colorectal cancer progression

**DOI:** 10.1007/s00535-026-02430-4

**Published:** 2026-04-27

**Authors:** Shang-Yin Wu, Po-Chuan Chen, Ren-Hao Chan, Chung-Hsing Chen, Yi-Hsuan Huang, Yi-Jing Huang, Bing-Syuan Chung, Che-Hung Shen, Hsin-Yu Kuo, Jui-Wen Kang, Chung-Ta Lee, Hui-Ju Tsai, Yu-Chen Fang, Peng-Chan Lin, Yu-Min Yeh, Shang-Hung Chen

**Affiliations:** 1 Department of Oncology, National Cheng Kung University Hospital, College of Medicine, National Cheng Kung University, No. 1, University Road, Tainan City, Taiwan; 2 Division of Colorectal Surgery, Department of Surgery, National Cheng Kung University Hospital, College of Medicine, National Cheng Kung University, No. 1, University Road, Tainan City, Taiwan; 3 Department of Mathematics, University of Taipei, No. 1, Aiguo West Road, Taipei City, Taiwan; 4 National Institute of Cancer Research, National Health Research Institutes, 2F, No. 367, Sheng Li Road, Tainan City, Taiwan; 5 Doctoral Program in Tissue Engineering and Regenerative Medicine, Biotechnology Center, National Chung Hsing University, No. 145, Xingda Road, Taichung City, Taiwan; 6 Center for Cancer Research, Kaohsiung Medical University, No. 100, Shih‑Chuan 1st Road, Kaohsiung City, Taiwan; 7 Department of Internal Medicine, National Cheng Kung University Hospital, College of Medicine, National Cheng Kung University, No. 1, University Road, Tainan City, Taiwan; 8 Department of Pathology, College of Medicine, National Cheng Kung University Hospital, National Cheng Kung University, No. 1, University Road, Tainan City, Taiwan; 9 Department of Genomic Medicine, College of Medicine, National Cheng Kung University Hospital, National Cheng Kung University, No. 1, University Road, Tainan City, Taiwan

**Keywords:** Single-cell RNA sequencing, Colorectal cancer, Tumor-associated macrophage, Cancer-associated fibroblast, TREM1, SPP1

## Abstract

**Background:**

The limited efficacy of immunotherapy in colorectal cancer (CRC) underscores the need to better define immunosuppressive mechanisms within the tumor microenvironment (TME). We applied single-cell RNA sequencing (scRNA-seq) to characterize stromal–immune interactions shaping the CRC TME.

**Methods:**

Paired tumor and adjacent normal mucosa samples from eight patients with CRC were analyzed by scRNA-seq. Integrated bioinformatic approaches, including trajectory inference, transcriptional regulon analysis, and cell–cell communication modeling, were used to define cellular differentiation and intercellular signaling. Key findings were validated using The Cancer Genome Atlas datasets, multiplex immunofluorescence staining, and in vitro functional assays.

**Results:**

Trajectory analysis demonstrated a progressive increase in triggering receptor expressed on myeloid cells 1 (TREM1) expression during tumor-associated myeloid differentiation. TREM1-positive myeloid cells exhibited enriched M2-like signatures and were preferentially associated with consensus molecular subtype 4 tumors, characterized by stromal activation and poor clinical outcomes. Stromal profiling identified an α-smooth muscle actin (ACTA2)-positive population associated with CRC progression. Spatial analyses revealed close localization of TREM1-positive myeloid cells and ACTA2-positive cancer-associated fibroblasts (CAFs) in tumor tissues. Mechanistically, integrated bioinformatic analyses indicated that TREM1-positive myeloid cells engage CAFs primarily through secreted phosphoprotein 1 (SPP1) signaling, while CAFs reinforce immunosuppressive myeloid phenotypes via TGF-β-associated extracellular matrix pathways. Functional assays showed that TREM1 inhibition reduced SPP1 expression and attenuated M2 macrophage polarization.

**Conclusions:**

These findings identify a bidirectional interaction between TREM1-positive myeloid cells and CAFs that contributes to a profibrotic and immunosuppressive CRC microenvironment, highlighting the TREM1–SPP1 axis as a pathway of potential translational relevance.

**Supplementary Information:**

The online version contains supplementary material available at 10.1007/s00535-026-02430-4.

## Background

Colorectal cancer (CRC) is a leading cause of cancer-related death worldwide [1, 2]. While advances in surgery and systemic therapy have improved outcomes, immune checkpoint inhibitors (ICIs) remain largely ineffective in microsatellite-stable (MSS) CRC, which constitutes most cases [3, 4]. Understanding the mechanisms that drive immune evasion in the tumor microenvironment (TME) is essential for improving therapeutic outcomes [5, 6]. The TME comprises malignant cells, stromal components, and diverse immune populations that collectively shape tumor progression and treatment response. Among these, tumor-associated myeloid cells (TAMCs)—including macrophages, dendritic cells, and neutrophils—represent a dominant and immunologically influential cell population [7, 8]. In many solid tumors, myeloid cells acquire an immunosuppressive, M2-like phenotype characterized by secretion of cytokines such as IL-10 and TGF-β, high expression of PD-L1, and the promotion of angiogenesis, invasion, and metastasis [9]. Through these mechanisms, TAMCs play a central role in suppressing cytotoxic T-cell activity and contribute substantially to resistance to ICIs in MSS CRC.

Cancer-associated fibroblasts (CAFs) represent another critical stromal component of the TME. CAFs promote tumor growth and therapy resistance through the secretion of cytokines and extensive remodeling of the extracellular matrix (ECM) [10, 11]. By producing structural components, such as collagen, laminin, fibronectin, and hyaluronic acid, CAFs generate a dense desmoplastic stroma that impedes immune cell infiltration and limits drug delivery [12, 13]. Notably, CAF-derived factors can recruit circulating monocytes and promote their differentiation into M2-like tumor-associated macrophages (TAMs), thereby reinforcing an immunosuppressive tumor milieu [14]. Recent advances in high-throughput technologies, particularly single-cell RNA sequencing (scRNA-seq), have enabled high-resolution characterization of cellular heterogeneity and intercellular communication within the TME [15, 16]. Emerging evidence suggests that CAF–TAMC crosstalk regulates multiple facets of tumor biology, including tumor growth, metastasis, angiogenesis, and immune evasion [17–19]. However, the specific mechanisms underlying CAF–TAMC interactions in MSS CRC remain poorly defined.

In the present study, we performed scRNA-seq on paired tumor and adjacent normal tissues from patients with CRC to comprehensively characterize the TME. We identified a distinct population of triggering receptor expressed on myeloid cells 1 (TREM1)-positive myeloid cells that were markedly enriched in tumor tissues and late-stage differentiated TAMCs. In parallel, we characterized α-smooth muscle actin (ACTA2)-positive CAFs displaying TGF-β-driven activation programs associated with CRC progression. Through integrated transcriptional regulon analysis and ligand–receptor interaction modeling, we identified secreted phosphoprotein 1 (SPP1) signaling from TREM1-positive myeloid cells to ACTA2-positive CAFs as a potential axis of stromal–immune crosstalk. TREM1, a member of the immunoglobulin superfamily, is a key amplifier of inflammatory responses in both acute and chronic inflammatory conditions [20, 21]. Although TREM1-positive macrophages have been implicated in the progression of several malignancies, their protumorigenic role in CRC remains insufficiently defined. Moreover, ACTA2-positive CAFs and SPP1-positive macrophages have each been reported to contribute to CRC progression and the establishment of an immunosuppressive TME [10, 11, 22]. However, the functional interplay among TREM1-positive myeloid cells, ACTA2-positive CAFs, and SPP1 signaling has not been fully elucidated. Therefore, this study aims to investigate the interactions between TREM1-positive myeloid cells and ACTA2-positive CAFs, with a particular focus on the role of SPP1 signaling in shaping CRC-associated immune and stromal remodeling.

## Methods

### Collection of clinical human samples

This study was conducted in accordance with the ethical principles of the Declaration of Helsinki. All participants provided written informed consent, and the study protocol was approved by the institutional ethics committee (approval numbers: B-BR-106-068 and A-ER-114-001). Adjacent normal mucosa and tumor tissues were collected from eight patients with CRC at National Cheng Kung University Hospital (NCKUH) between June and December 2022. Freshly resected specimens were immediately transferred to RPMI-1640 medium (ThermoFisher Scientific, Waltham, MA, USA) supplemented with 10% fetal bovine serum (FBS) and transported on ice for downstream processing. Genomic profiling and microsatellite status were determined using a TruSight Oncology 500 assay (Illumina, San Diego, CA, USA).

### Tissue dissociation and single-cell suspension preparation

Fresh tissues were enzymatically dissociated using a Whole Skin Dissociation Kit (Miltenyi Biotec, Bergisch Gladbach, Germany) at 37 °C for 1 h with intermittent mixing. The digested samples were diluted in DMEM (ThermoFisher Scientific, Waltham, MA, USA) supplemented with 10% FBS and gently resuspended. Cell suspensions were filtered through a 40-μm EasyStrainer (Greiner, Frickenhausen, Germany) and centrifuged at 1200 rpm for 5 min at 4 °C. Pellets were treated with 1 × red blood cell lysis buffer for 2 min at room temperature, washed, and resuspended in cold DMEM containing 10% FBS. Cell number and viability were assessed using a TC20 automated cell counter (Bio-Rad, Hercules, CA, USA) prior to scRNA-seq library preparation.

### scRNA-seq library preparation

Libraries were prepared using Chromium Single Cell 3′ Reagent Kits v3.1 (10 × Genomics, Pleasanton, CA, USA) in accordance with the manufacturer’s protocol. Briefly, approximately 13,000 cells per sample were mixed with reverse transcription reagents and loaded into a Chromium Next GEM Chip G, along with Single Cell 3′ v3.1 Gel Beads and partitioning oil. The chip was processed on a Chromium Controller to generate gel bead-in-emulsions. The targeted recovery was approximately 7,000 cells per sample, with an estimated multiplet rate of 6%. Libraries were constructed following the manufacturer’s instructions, quantified using a Qubit dsDNA HS Assay Kit (Thermo Fisher Scientific), and evaluated for integrity and average fragment size by using an Agilent 4150 TapeStation system. The final libraries were sequenced on a NovaSeq 6000 platform (Illumina).

### ScRNA-seq and data processing

Fresh single-cell suspensions were processed using the Chromium Single Cell 3′ v3 platform (10 × Genomics) according to the manufacturer’s instructions. Libraries were prepared and sequenced on an Illumina NovaSeq 6000 system. Base calling and FASTQ generation were performed using bcl2fastq2 (v2.20), and read quality was assessed with FastQC (v0.11.9). Reads were aligned to the GRCh38 reference genome and quantified using the Cell Ranger pipeline (10 × Genomics) with default settings. Downstream quality control, normalization, and filtering were conducted using Seurat (v4.3.0), retaining cells with > 1000 UMIs, 200–6000 detected genes, and < 20% mitochondrial gene content, as previously described [23].

### Data integration, dimension reduction, and clustering analysis

In accordance with the standard Seurat workflow and data integration pipeline, we performed feature selection for each sample by using the *FindVariableFeatures()* function. Data integration was conducted using the *FindIntegrationAnchors()* and *IntegrateData()* functions. The integrated dataset was scaled using the *ScaleData()* function and projected into the first 50 principal components (PCs) by using the *RunPCA()* function. On the basis of the *ElbowPlot()* output, most biological variance was captured within the first 30 PCs. Graph-based Louvain clustering was performed on the top 30 PCs by using the *FindNeighbors()* and *FindClusters()* functions across a range of resolution parameters (0.1–1.0). Dimensionality reduction for visualization was performed using the *RunUMAP()* function, and the umap-learn algorithm with Pearson correlation was used as the distance metric. For cell-type annotation, the pre-labeled dataset GSE132465 was used as the reference. The SingleR algorithm (v1.10.0) [24] was applied to transfer labels to the MSS CRC dataset in accordance with the standard workflow. Clusters identified at a resolution of 0.2 were subsequently renamed in accordance with SingleR-derived annotations. Single-cell RNA-seq data were visualized using the *dittoBarPlot()* function from the dittoSeq package (version 1.8.1).

### Trajectory reconstruction

To reconstruct lineage relationships among single cells, we applied Monocle 3 (v1.0.0) to the processed scRNA-seq data. Cells from the myeloid population were subsetted in Seurat (version 4.3.0) and converted into a Monocle object by using the *as.cell_data_set()* function [25]. The trajectory graph was generated using the *learn_graph()* function, and pseudotime ordering was performed using *order_cells()*. The root state was defined as the cluster predominantly enriched in adjacent normal tissues. Pseudotime distributions were visualized using ggplot2 (version 3.5.0).

### UCell scoring of macrophage state signatures

To evaluate macrophage functional states at a single-cell level, we used the UCell algorithm implemented in the *escape* R package (v1.6.0). Gene signature sets for nine macrophage states (MS; MS1–MS9) were obtained from the Carcinoma EcoTyper framework, as described by Luca et al. [17]. For each cell, UCell scores were calculated on the basis of the relative enrichment of signature genes by using the default *enrichIt (method* = *“UCell”)* function. Signatures MS1–MS3 were grouped as M1-like states, and MS4–MS9 were grouped as M2-like states. Signature scores were visualized across clusters and tissue origins by using ggpubr (version 0.4.0).

### Immunofluorescence staining

Immunofluorescence staining was performed on 4-μm-thick formalin-fixed, paraffin-embedded tissue sections. Sections were deparaffinized in xylene, rehydrated through a graded ethanol series (100%–70%), and subjected to antigen retrieval by using 1 × retrieval buffer (Dako, Glostrup, Denmark) in an autoclave. Endogenous peroxidase activity was quenched using 3% hydrogen peroxide. Slides were incubated overnight at 4 °C in a humidified chamber with the following primary antibodies: TREM1 (1:100, conjugated to fluorescent dye 568), ACTA2 (1:200), epithelial cell adhesion molecule (EpCAM, 1:100), and CD163 (1:100, conjugated to fluorescent dye 488). After washing, sections were mounted with ProLong Gold Antifade Reagent and coverslipped. Fluorescence images were acquired using a confocal laser scanning microscope (FV1000; Olympus, Tokyo, Japan) and analyzed with Imaris software (version 7.2.3). Detailed information on antibodies and reagents is provided in Supplementary Table 1.

### Hallmark gene set enrichment analysis

To investigate cancer-related biological programs, we performed gene set enrichment analysis (GSEA) by using the hallmark gene sets curated in the Molecular Signatures Database (MSigDB, v2025.1). For each cell, enrichment scores of the 50 hallmark pathways were calculated using the UCell algorithm implemented in the *escape* package. For comparisons between cell populations, pathway activity scores were analyzed using the Wilcoxon rank-sum test with Benjamini–Hochberg correction for multiple testing. A false discovery rate (FDR) of < 0.2 was considered statistically significant.

### Bulk RNA-seq and immune infiltration analyses

Transcriptomic data, clinical annotations, and consensus molecular subtype (CMS) classifications for colorectal cancer were obtained from The Cancer Genome Atlas (TCGA) Colon and Rectal Cancer (COADREAD) cohort through the UCSC Xena platform, cBioPortal, and the Synapse repository, respectively [26, 27]. Immune and stromal cell proportions were estimated using the EPIC deconvolution algorithm implemented in TIMEDB. Specific associations between gene expression and immune cell infiltration were further evaluated using TIMER 2.0. Differential gene expression and clinicopathological correlation analyses were performed using the UALCAN platform. Spearman’s rank correlation coefficients were calculated to assess relationships between gene expression levels and immune or stromal features.

### Spatial transcriptome dataset

The human CRC spatial transcriptomic dataset was obtained from the 10 × Genomics website (https://www.10xgenomics.com/datasets/human-colorectal-cancer-11-mm-capture-area-ffpe-2-standard; accessed September 11, 2025). Standard procedures were followed to integrate the scRNA-seq data with the spatial transcriptomic data. After integration, cell-type prediction scores were visualized using the *SpatialFeaturePlot()* function.

### Transcription factor regulon activity analysis

Transcription factor (TF) activity at single-cell resolution was inferred using the *decoupleR* package (version 2.9.7). CollecTRI-derived regulons were obtained using the *get_collectri()* function, and TF activity scores were calculated for each cell by using the univariate linear model (*run_ulm()* function). The top 100 TFs exhibiting the highest variability across clusters were selected to generate the heatmap.

### Cell–cell communication analysis

Cell–cell communication networks were inferred using the *CellChat* package (version 1.6.1) with processed scRNA-seq data, annotated cell types, and the default ligand–receptor database (*CellChatDB.human*). Standard analyses, including *identifyOverExpressedGenes()*, *computeCommunProb()*, and *computeCommunProbPathway()*, were applied. The resulting networks were visualized using circle plots (*netVisual_circle()*), chord diagrams (*netVisual_chord_gene()*), ligand–receptor contribution analyses (*netAnalysis_contribution()*), and communication probability heatmaps generated with *Morpheus*.

### Cell culture and treatment

Human monocytic leukemia THP-1 cells (Bioresource Collection and Research Center, Hsinchu, Taiwan) were cultured in RPMI-1640 medium supplemented with 10% FBS and 1 × penicillin–streptomycin–glutamine (100 U/mL penicillin, 100 μg/mL streptomycin, and 2 mM L-glutamine). For differentiation into macrophages and subsequent M2 polarization, THP-1 cells were pre-treated with 50 ng/mL 12-O-tetradecanoylphorbol-13-acetate for 6 h, followed by incubation with 40 ng/mL IL-4 and 40 ng/mL IL-13 for 24 h. For pharmacological experiments, M2-polarized macrophages were treated with the TREM1 inhibitor VJDT for 24 h. Non-transformed human colon fibroblasts (CCD-18Co, ATCC, Manassas, VA, USA) were cultured in Eagle’s Minimum Essential Medium (EMEM) supplemented with 10% FBS under standard conditions. For coculture experiments, CCD-18Co fibroblasts and THP-1-derived macrophages were cultured using a Transwell system with 0.4 μm pore-size inserts (Corning, NY, USA), allowing paracrine interactions without direct cell contact. Fibroblasts were seeded in 6-well plates, while an equal number of THP-1-derived macrophages were seeded in the corresponding Transwell inserts. Prior to coculture, the medium in both compartments was replaced with EMEM to ensure consistency. Cells were cocultured for 24–48 h before downstream analyses. Detailed information on all reagents is provided in Supplementary Table 1.

### Short hairpin RNA transfection

Lentiviral particles were produced by cotransfecting HEK293T cells with pLKO.1 vectors encoding TREM1 shRNA (TRCN0000056753 or TRCN0000056755; RNAi Core, Academia Sinica, Taipei, Taiwan) or control shRNA using Effectene (Qiagen, Venlo, The Netherlands). Viral supernatants were collected 48 h post-transfection, centrifuged, and filtered (0.45 μm). Target cells were infected with viral supernatants containing 5 μg/mL polybrene for 6 h, followed by medium replacement. A second infection was performed the next day. Stable transductants were selected with puromycin (2 μg/mL) for 48–72 h prior to downstream analyses.

### Quantitative real‑time PCR

Cells were lysed in TRIzol reagent (Invitrogen Life Technologies, Carlsbad, CA, USA), and total RNA was extracted using Qiagen spin columns (Qiagen, Valencia, CA, USA). Complementary DNA was synthesized with the SuperScript First Strand Synthesis System (Invitrogen Life Technologies). Primers for M2 macrophage and CAF markers are listed in Supplementary Table 1. The relative mRNA expression of target genes was measured using the ABI 7500 Sequence Detection System (Applied Biosystems, Waltham, MA, USA) and analyzed using the ΔΔCt method, with GAPDH serving as the endogenous control.

### Western blot analysis

Cells were lysed using lysis buffer (Merck Millipore, Darmstadt, Germany), and total proteins were extracted and separated by sodium dodecyl sulfate–polyacrylamide gel electrophoresis. Proteins were subsequently transferred onto polyvinylidene fluoride membranes (Merck Millipore). Immunoreactive bands were detected using the Western Lightning Plus-ECL Enhanced Chemiluminescence Substrate (PerkinElmer, Waltham, MA, USA) and visualized on Kodak X-Omat film (Kodak, Paris, France). The following primary antibodies were used: TREM1 (1:2000), DNAX activating protein of 12 kDa (DAP12; 1:1000), SPP1 (1:1000), FAP (1:1000), PDGFR-α (1:1000), and GAPDH (1:10,000). Detailed information on all antibodies is provided in Supplementary Table 1.

### Statistical analysis

Survival analyses were conducted using the Kaplan–Meier method with log-rank tests (*survival* v3.3—1, *survminer* v0.4.9), optimal cutpoints identified with *surv_cutpoint()*, and Cox proportional hazards models (*finalfit* v1.0.7) to estimate hazard ratios with 95% confidence intervals. Gene expression correlations were evaluated using Spearman’s coefficient (*Hmisc* v4.7—1, *corrplot* v0.92), and comparisons among groups were conducted using Wilcoxon–Mann–Whitney or Kruskal–Wallis tests. Data visualizations were generated as Venn diagrams (*Venny*), heatmaps (*Morpheus*), box and scatter plots (*ggpubr* v0.4.0), and Kaplan–Meier curves (*ggsurvplot()*). All statistical analyses were performed using GraphPad Prism (version 8.0.1; Boston, MA, USA) and *R* (version 4.2.1). All tests were two-sided, and a *P* value of < 0.05 was considered statistically significant.

## Results

### Immune landscape characterization of CRC

**Fig. 1:**
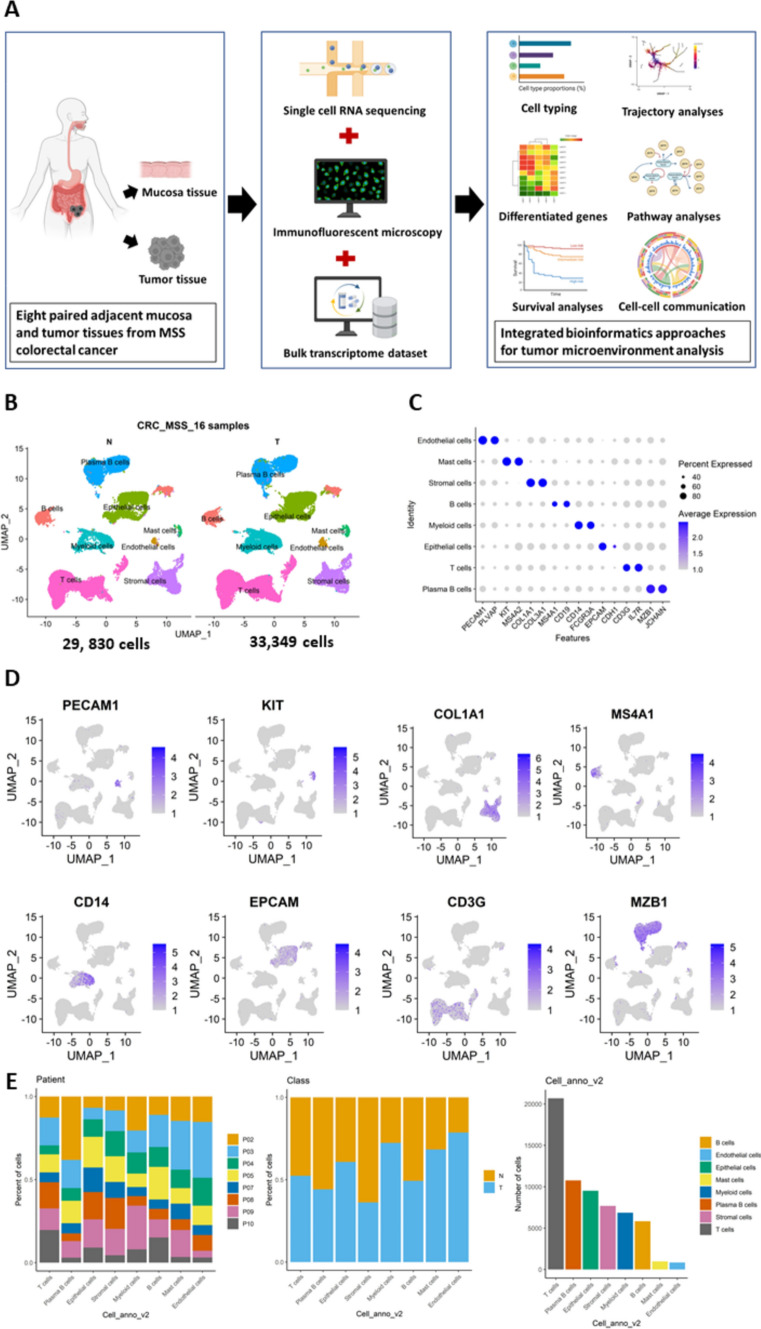
Single-cell atlas of paired normal mucosa and CRC tissues. **A** Schematic overview of the study. Normal mucosa and tumor tissues from eight patients with MSS CRC were dissociated into single-cell suspensions and subjected to scRNA-seq by using the 10 × Genomics platform. Tumor sections were analyzed using immunofluorescence staining to validate bioinformatic findings. Additional validation was performed using the TCGA COADREAD dataset and other publicly available datasets. **B** UMAP plots of 29,830 cells from normal mucosa and 33,349 cells from tumor tissues across eight patients, integrated using the CCA algorithm. Eight cell types are represented as separate clusters, each depicted in a different color. **C** Dot plots depicting the average expression of established marker genes across the identified cell types. Dot size indicates the proportion of cells expressing each gene within a given cell type, and color intensity represents the average expression level. **D** UMAP plots illustrating the expression of previously reported marker genes across 63,179 cells derived from both normal and tumor tissues of patients with MSS CRC. **E** Proportions of the eight identified cell types, presented as bar plots stratified by patient (left panel) and tissue type (middle panel), with total cell counts for each cell type depicted in the right panel. *N* normal, *T* tumor, *CRC* colorectal cancer, *scRNA-seq *single-cell RNA sequencing, *TCGA COADREAD* The Cancer Genome Atlas colorectal adenocarcinoma, *UMAP* uniform manifold approximation and projection, *CCA* canonical correlation analysis

To investigate tumor immunity in MSS CRC, we performed scRNA-seq on paired tumor and adjacent normal mucosa from eight patients receiving surgery at NCKUH. Clinical and genomic characteristics are summarized in Supplementary Table 3, and the study workflow is presented in Fig. 1A. After stringent quality filtering to remove low-quality cells and doublets, 63,179 high-confidence transcriptomes were retained, including 29,830 cells from normal mucosa and 33,349 cells from tumors (Fig. 1B). Unsupervised clustering and cell-type annotation using SingleR (reference dataset GSE132465) identified eight major cell populations (Fig. 1B–D): epithelial cells, T cells, B cells, plasma cells, myeloid cells, mast cells, endothelial cells, and stromal cells, consistent with established markers [25]. Compared with normal mucosa, tumor tissues exhibited higher proportions of myeloid and endothelial cells, highlighting an immunologically remodeled TME (Fig. 1E, left and middle). T cells were the most abundant population overall, followed by plasma cells, stromal cells, and myeloid cells (Fig. 1E, right).

### Characterization of myeloid cells in the normal mucosa and tumor tissue

**Fig. 2:**
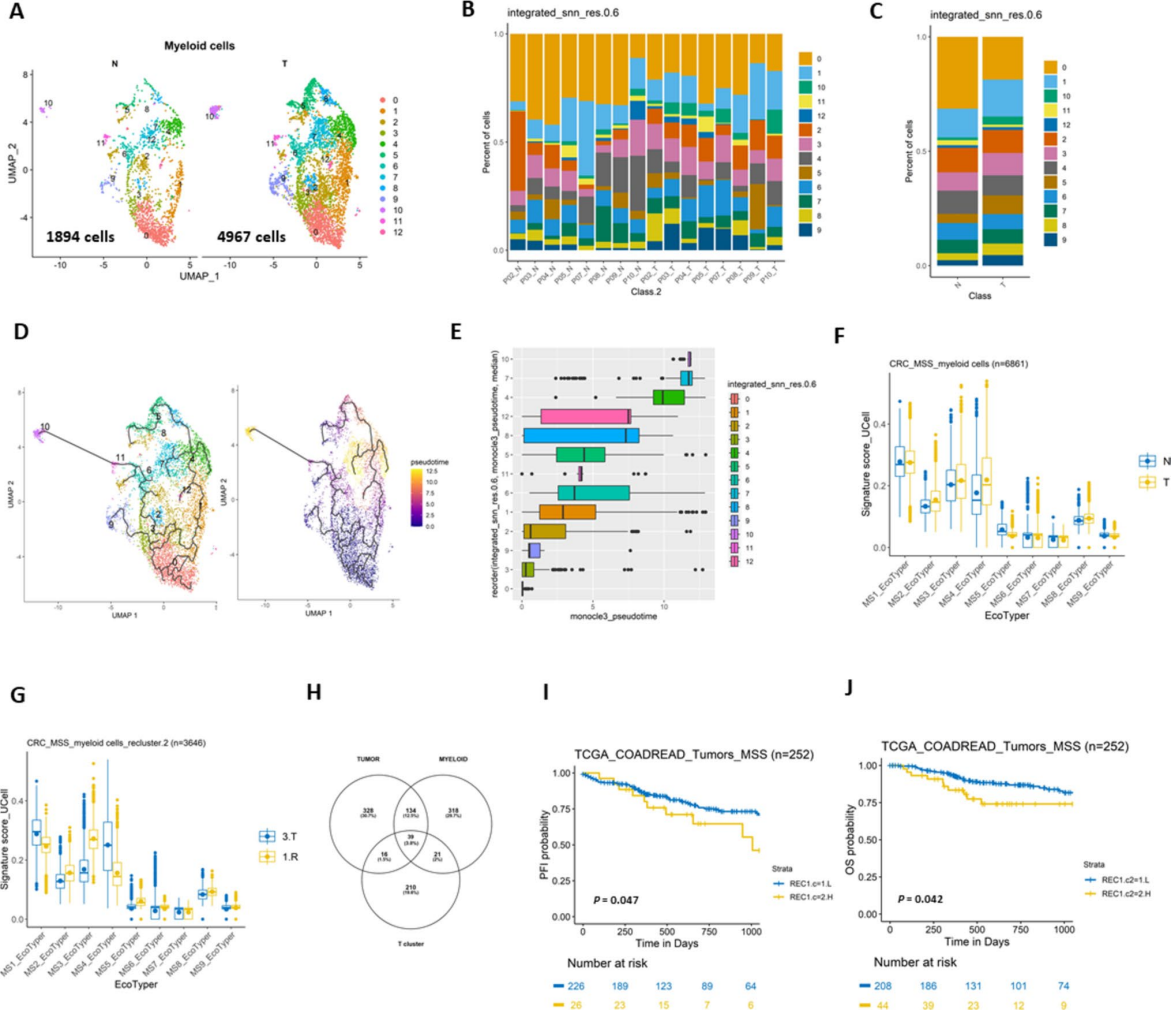
Terminally differentiated myeloid cells are correlated with tumor-associated macrophages and survival outcomes in CRC. **A** Subclustering analysis of 6861 myeloid cells (*n* = 8 patients; clusters 0–12). Of these, 1894 cells were derived from normal tissues (N), and 4967 cells were derived from tumor tissues (T). **B**, **C** Bar plots depicting the proportions of each myeloid cluster across individual samples (**B**) and aggregated by tissue type (normal vs. tumor) (**C**). **D** Trajectory analysis of myeloid cells inferred using the Monocle3 algorithm. Left panel: UMAP visualization of the myeloid cell trajectory (colored as in **A**). Right panel: UMAP visualization of the trajectory colored by pseudotime. **E** Box plots depicting Monocle3-inferred pseudotime values across the 13 myeloid clusters. **F** Box plots depicting signature scores of myeloid cells (*n* = 6861) from normal (N) and tumor (T) tissues across the nine *EcoTyper* macrophage states. **G** Box plots depicting the signature scores of myeloid cells (*n* = 3646) from Root (R; cluster 0) and Terminal (T; clusters 4, 7, and 10) populations across the nine *EcoTyper* macrophage states. **H** Venn diagram depicting the number of genes enriched in tumor tissues (vs. normal tissues), myeloid cells (vs. other cell types), and terminal myeloid cells (clusters 4, 7, and 10; vs. other myeloid clusters). **I**, **J** Kaplan–Meier curves for progression-free interval (**I**) and overall survival (**J**) of TCGA COADREAD MSS tumor samples (*n* = 252), stratified by REC1 gene signature score. The log-rank *P* value is indicated in each plot. *CRC* colorectal cancer, *UMAP* uniform manifold approximation and projection, *TCGA COADREAD* The Cancer Genome Atlas colorectal adenocarcinoma, *MSS* microsatellite stable, *PFI* progression-free interval, *OS* overall survival, *H* high, *L* low

Given the enrichment of myeloid cells in tumor tissues, we performed a focused analysis of this compartment. Reclustering of 6861 myeloid cells identified 13 transcriptionally distinct subsets at a resolution of 0.6 (Fig. 2A). Among these, cluster 0 was predominantly enriched in adjacent normal tissues (Fig. 2B, C), suggesting that it represents a progenitor-like root state. Trajectory inference using *Monocle3* designated cluster 0 as the root, with clusters 4, 7, and 10 mapping to terminally differentiated states within the TME (Fig. 2D, E). To functionally characterize these subsets, we applied *UCell* scoring to nine MS signatures from the Carcinoma *EcoTyper* framework, grouping MS1–MS3 as M1-like states and MS4–MS9 as M2-like states. Tumor-associated myeloid cells exhibited significantly higher M2-like (MS4) scores compared with myeloid cells from adjacent normal tissues (Fig. 2F). Further comparison revealed that the root population (R; cluster 0) was enriched for the proinflammatory MS3 signature, whereas the terminal populations (T; clusters 4, 7, and 10) were enriched for the immunosuppressive MS4 signature (Fig. 2G). To identify tumor-specific effectors, we intersected marker genes from tumor-derived cells, the global myeloid compartment, and terminal clusters, generating a tumor-associated myeloid signature designated REC1 (Fig. 2H). In the TCGA COADREAD cohort (*n* = 252), high REC1 expression was associated with significantly shorter progression-free interval (PFI; Fig. 2I) and overall survival (OS; Fig. 2J).

### TREM1-positive myeloid cells are associated with M2 macrophages and survival outcomes of CRC

**Fig. 3:**
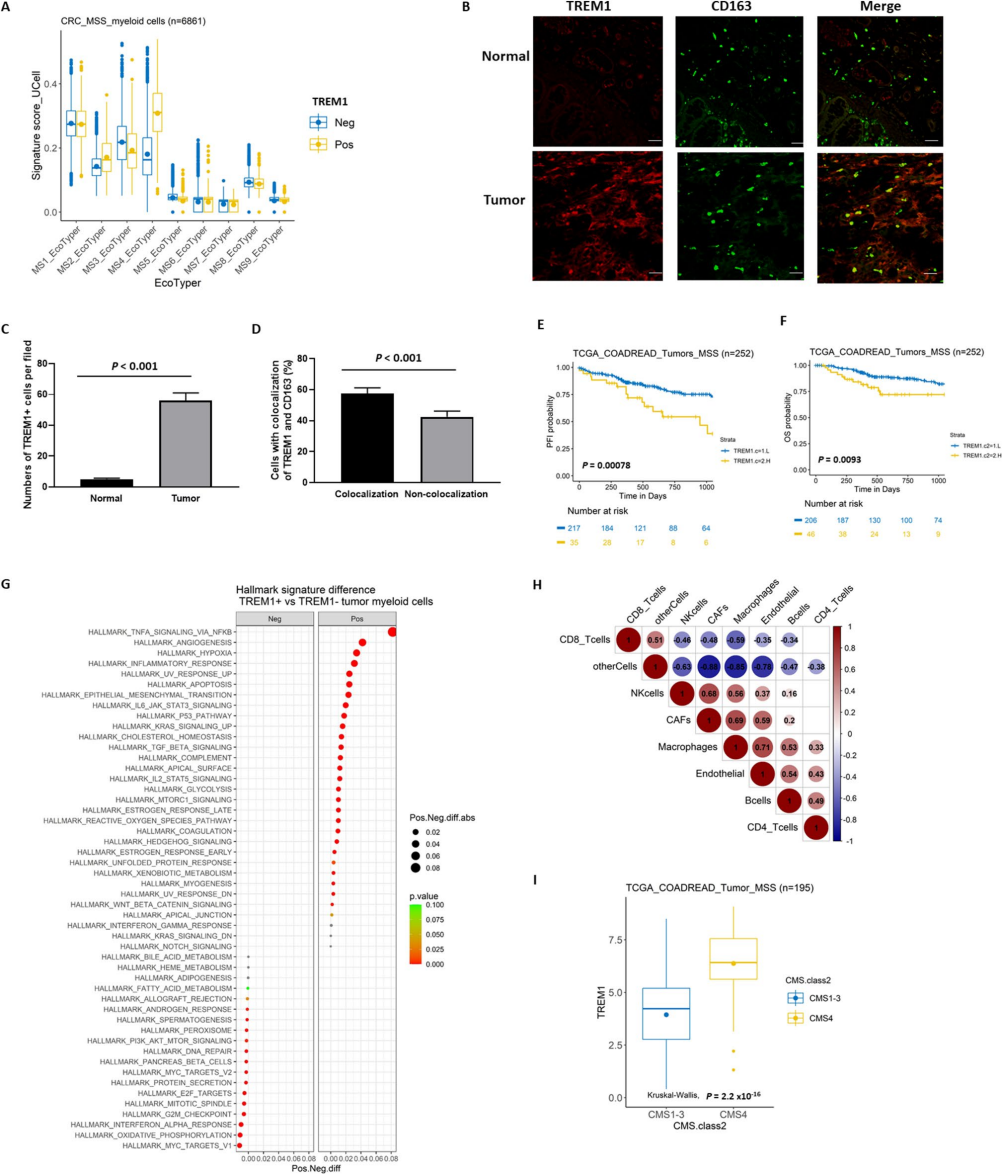
TREM1-positive myeloid cells are correlated with M2 macrophage polarization and survival outcomes in CRC. **A** Box plots depicting signature scores of TREM1-positive (Pos) and TREM1-negative (Neg) myeloid cells across the nine *EcoTyper*-defined macrophage states. **B** Representative immunofluorescence staining of human CRC tissue (20 × magnification), exhibiting CD163 (green) and TREM1 (red) in individual and merged channels. Scale bar = 50 μm. **C**, **D** Quantification of TREM1-positive cells in normal versus tumor tissues (**C**) and their colocalization with CD163-positive cells (**D**) across 30 tissue sections from five patients. **E**, **F** Kaplan–Meier curves for progression-free interval (**E**) and overall survival (**F**) of TCGA COADREAD MSS tumor samples (*n* = 252), stratified by TREM1-low and TREM1-high expression groups. Log-rank *P* values are shown in each plot. (**G**) Dot plot depicting differences in hallmark gene signature scores calculated using the *UCell* algorithm between TREM1-positive and TREM1-negative myeloid cells derived from tumor tissues. (**H**) Correlation matrix illustrating cell type proportions in TCGA COADREAD MSS tumor samples (*n* = 177) estimated using *EPIC* deconvolution. Only correlations with *P* < 0.05 are depicted. Spearman correlation coefficients are indicated within the plot, with red denoting positive and blue denoting negative correlations. (**I**) Box plots depicting TREM1 expression levels in TCGA COADREAD MSS tumor samples (*n* = 195), comparing CMS4 with CMS1–3 subtypes. Statistical significance was assessed using the Kruskal–Wallis test. *TREM1* triggering receptor expressed on myeloid cells 1, *CRC *colorectal cancer, *TCGA COADREAD* The Cancer Genome Atlas colorectal adenocarcinoma, *MSS* microsatellite stable, *PFI* progression-free interval, *OS* overall survival, *H* high, *L* low, *CMS* consensus molecular subtype

Given the association between the REC1 gene signature and adverse clinical outcomes in CRC, we next sought to identify key regulators of tumor-associated myeloid programs. Comparative analysis of genes enriched in tumor-derived myeloid cells and terminal differentiation clusters revealed TREM1 as the most significantly upregulated gene in tumors with poor prognosis in the TCGA cohort (Supplementary Table 4). As TREM1 is a known amplifier of myeloid inflammatory signaling [20, 21], we classified cells as TREM1-positive or TREM1-negative based on raw count detection. TREM1-positive myeloid cells demonstrated significantly higher M2-like macrophage scores (MS4) and lower M1-like (MS3) scores than TREM1-negative cells (Fig. 3A). Immunofluorescence staining confirmed minimal TREM1 expression in normal mucosa and an approximately 6.3-fold enrichment in tumor tissues (Fig. 3B–D). Co-staining with CD163, a canonical M2 macrophage marker [28], revealed that roughly 60% of TREM1-positive cells were CD163-positive (Fig. 3D), indicating predominance of M2-like phenotypes. Clinically, high TREM1 expression in the TCGA cohort was associated with significantly poorer PFI and OS (Fig. 3E, F). Transcriptomic pathway analyses showed significant enrichment of oncogenic signatures in TREM1-positive cells, including TNF-α/NF-κB signaling, epithelial–mesenchymal transition (EMT), and MAPK signaling pathway (Fig. 3G and Supplementary Fig. 1A). Given that EMT is largely driven by CAFs [29, 30], we further explored the relationship between myeloid and stromal components. Infiltration analyses demonstrated a strong correlation between macrophage and CAF abundance (Spearman *r* = 0.69, *P* < 0.05; Fig. 3H). Among samples with CMS classification, TREM1 expression was significantly higher in CMS4 tumors—known for stromal activation and poor prognosis [31, 32]—than in other CMS subtypes (Fig. 3I).

Analysis of TCGA immune transcriptomic data showed that TREM1 expression was markedly elevated in CRC compared with normal colon tissue (Supplementary Fig. 2A). TREM1 levels correlated positively with M2 macrophage abundance (Supplementary Fig. 2B) and CAF infiltration (Supplementary Fig. 2C) but negatively with CD8⁺ T-cell levels (Supplementary Fig. 2D). Notably, late-stage differentiated effector and exhausted effector memory CD8^+^ T cell states, assigned via Carcinoma EcoTyper, exhibited significantly higher TREM1 expression compared to naive and central memory CD8^+^ T cell states in bulk TCGA datasets (Supplementary Fig. 2E). Survival analysis using the KM Plotter database further revealed that high TREM1 expression predicted significantly shorter OS in CRC (Supplementary Fig. 2F), highlighting its association with an immunosuppressive microenvironment and poor prognosis.

### ACTA2-positive CAFs promote tumor progression and immunosuppression in CRC

**Fig. 4:**
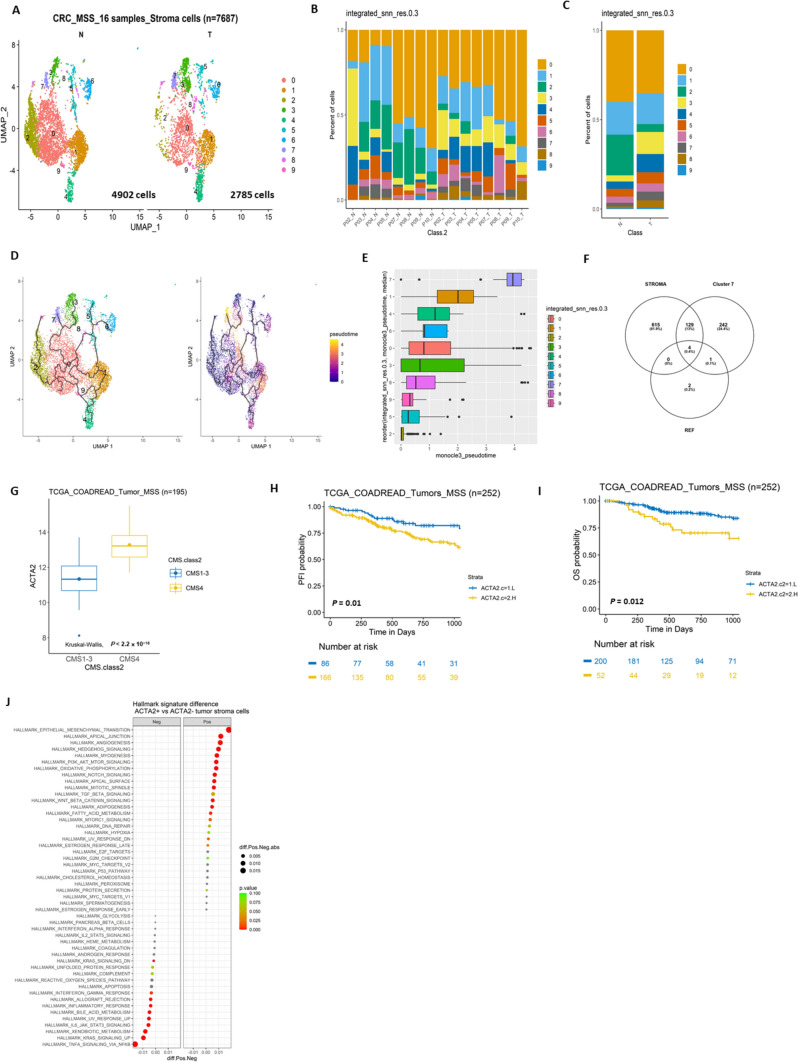
ACTA2-positive stromal cells are correlated with CAFs in CRC tissues. **A** Subclustering analysis of 7687 stromal cells (*n* = 8 patients; clusters 0–9). Of these, 4902 cells were derived from normal tissues (N), and 2785 cells were derived from tumor tissues (T). **B**, **C** Bar plots depicting the proportions of each stromal cluster across individual samples (**B**) and aggregated by tissue type (normal vs. tumor) (**C**). **D** Trajectory analysis of stromal cells. Left panel: UMAP visualization of the stromal cell trajectory path (colored as in **A**). Right panel: UMAP visualization of the trajectory colored by pseudotime. **E** Box plots depicting Monocle3-inferred pseudotime values across the 10 stromal clusters. **F** Venn diagram depicting the number of genes enriched in stromal cells (vs. other cell types), in cluster 7 stromal cells (vs. other stromal clusters), and those reported in the reference (REF). **G** Box plots depicting ACTA2 expression levels in TCGA COADREAD MSS tumor samples (*n* = 195), comparing CMS4 with CMS1–3 subtypes. Statistical significance was examined using the Kruskal–Wallis test. **H**, **I** Kaplan–Meier curves for PFI (**H**) and OS (**I**) of TCGA COADREAD MSS tumor samples (*n* = 252), stratified by ACTA2-low and ACTA2-high expression groups. Log-rank *P* values are indicated in each plot. (**J**) Dot plot depicting differences in hallmark gene signature scores calculated using the *UCell* algorithm between ACTA2-positive and ACTA2-negative stromal cells derived from tumor tissues. *ACTA2* α-smooth muscle actin, *CAF* cancer-associated fibroblast, *CRC* colorectal cancer, *UMAP* uniform manifold approximation and projection, *TCGA COADREAD* The Cancer Genome Atlas colorectal adenocarcinoma, *MSS* microsatellite stable, *PFI* progression-free interval, *OS* overall survival, *H* high, *L* low, *CMS* consensus molecular subtype

To investigate stromal contributions, we subdivided 7687 stromal cells into 10 transcriptionally distinct populations (resolution = 0.3; Fig. 4A). Cluster 2 was enriched in normal tissue and was designated the stromal root state (Fig. 4B, C). Trajectory analysis identified cluster 7 as a terminally differentiated fibroblast subset in the tumor (Fig. 4D, E). Comparative analysis of stromal gene signatures integrated with TCGA datasets identified ACTA2 as a defining marker of this CAF-enriched cluster (Fig. 4F; Supplementary Table 5). In the TCGA cohort, CMS4 tumors exhibited significantly higher ACTA2 expression than CMS1–3 tumors (Fig. 4G). High ACTA2 levels correlated with shorter PFI and OS (Fig. 4H, I). Within the scRNA-seq dataset, ACTA2-positive fibroblasts displayed increased EMT scores (Fig. 4J). Consistent with findings in TREM1-positive myeloid cells, the selected 23 KEGG signaling pathway enrichment analyses demonstrated a positive association between ACTA2-positive fibroblasts and MAPK signaling (Supplementary Fig. 1B). TCGA analyses further showed strong correlations between ACTA2 expression and CAF infiltration (Supplementary Fig. 3A) as well as M2 macrophage abundance (Supplementary Fig. 3B). Consistently, survival analysis performed using the KM Plotter database indicated that high ACTA2 expression was associated with significantly shorter OS in patients with CRC (Supplementary Fig. 3C).

### TREM1-positive myeloid cell–CAF interactions promote tumor progression and lead to poor prognosis in CRC

**Fig. 5:**
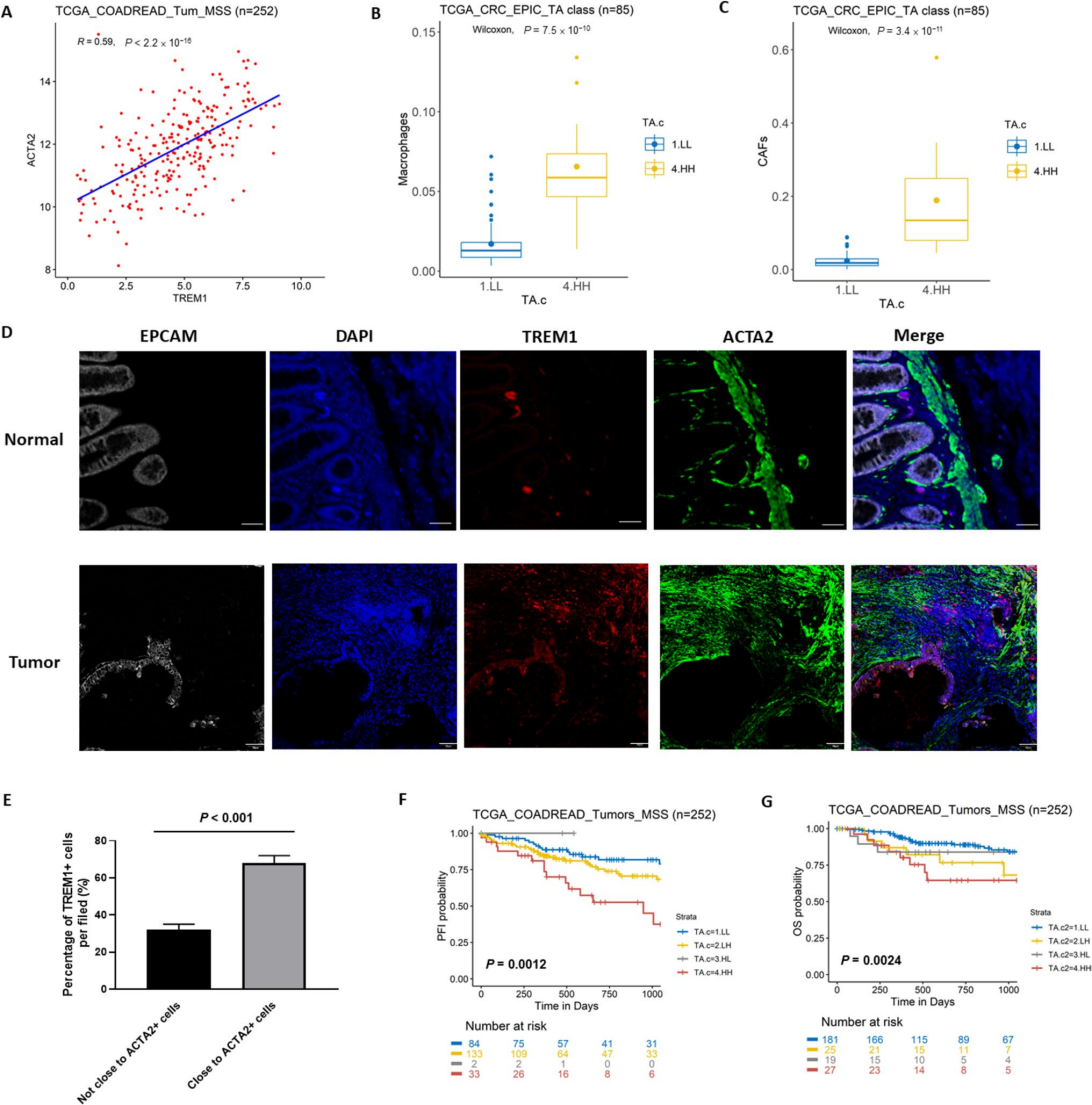
Spatial interaction between TREM1-positive myeloid cells and ACTA2-positive CAFs correlates with poor prognosis in CRC. **A** Scatter plot depicting the Spearman correlation between TREM1 and ACTA2 expression in TCGA COADREAD MSS tumor samples (*n* = 252). The correlation coefficient (R) and *P* value are indicated in the plot. **B**, **C** Box plots depicting the proportions of (**B**) macrophages and (**C**) CAFs in TCGA COADREAD MSS tumor samples with EPIC deconvolution data. Samples (*n* = 85) were stratified into LL and HH groups by using the same criteria applied in the PFI analysis. Statistical significance was examined using the Wilcoxon–Mann–Whitney test. **D** Representative immunofluorescence staining of human CRC tissues (20 × magnification), exhibiting EPCAM (gray), DAPI (blue), ACTA2 (green), and TREM1 (red) in individual and merged channels. Scale bar = 50 μm. **E** Proportion of TREM1-positive myeloid cells in physical contact with ACTA2-positive fibroblasts, quantified across 30 tissue sections from 5 patients. **F**, **G** Kaplan–Meier curves depicting **F** progression-free interval and **G** overall survival in TCGA COADREAD MSS tumor samples (*n* = 252), stratified by TREM1/ACTA2-high and TREM1/ACTA2-low groups. Group classification was based on the same criteria used for the progression-free interval analysis of each individual gene. *TREM1* triggering receptor expressed on myeloid cells 1, *ACTA2* α-smooth muscle actin, *CAF* cancer-associated fibroblast, *CRC* colorectal cancer, *TCGA COADREAD* The Cancer Genome Atlas colorectal adenocarcinoma, *MSS* microsatellite stable, *PFI* progression-free interval, *OS* overall survival, *LL* TREM1-low/ACTA2-low, *HH* TREM1-high/ACTA2-high

Given the associations observed in single-cell and bulk datasets, we next investigated the spatial and functional interaction between TREM1-positive myeloid cells and ACTA2-positive CAFs. In the TCGA cohort, TREM1 and ACTA2 expressions were strongly correlated (*R* = 0.59,* P* < 2.2 × 10^-16^; Fig. 5A). Tumors with high coexpression of TREM1 and ACTA2 exhibited significantly increased infiltration of macrophages and CAFs compared with other tumors (Fig. 5B, C). Multiplex immunofluorescence staining confirmed ACTA2 as a CAF marker and revealed that approximately 68% of ACTA2-positive CAFs were located in close proximity to TREM1-positive myeloid cells within tumor tissues, a spatial pattern that was not observed in adjacent normal mucosa (Fig. 5D, E). These findings were further supported by spatial transcriptomic analyses, which demonstrated concordant co-localization of TREM1-positive myeloid cells and ACTA2-positive stromal regions (Supplementary Fig. 4). Clinically, patients whose tumors exhibited high TREM1/ACTA2 coexpression had significantly shorter PFI and OS in the TCGA cohort (Fig. 5F, G).

### SPP1 signaling mediates crosstalk between TREM1-positive myeloid cells and CAFs in a desmoplastic TME

**Fig. 6:**
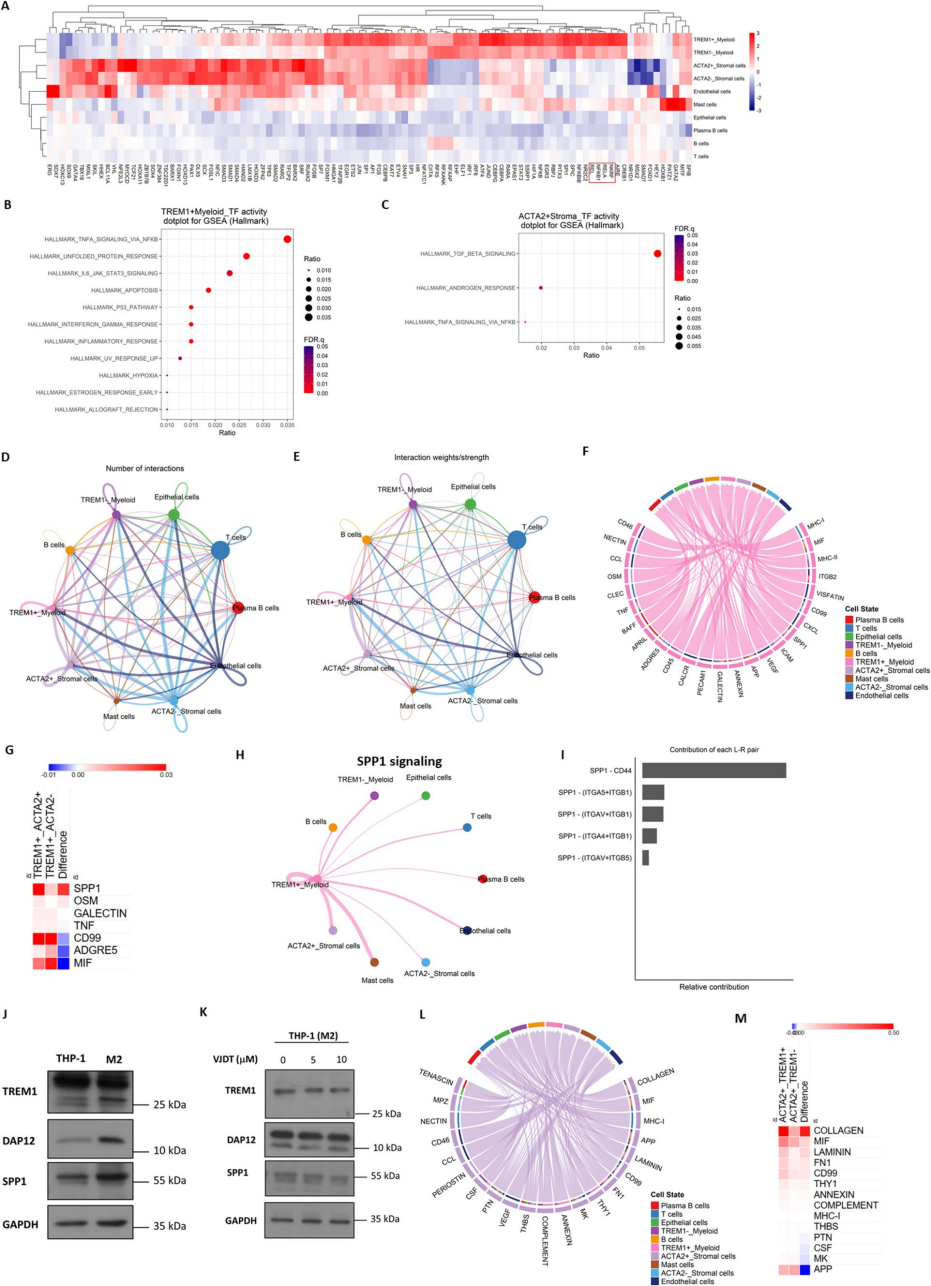
Interaction networks between TREM1-positive myeloid cells and CAFs in CRC. **A** scRNA-seq data from tumor tissues (*n* = 8) were analyzed using the decoupleR algorithm to infer TF activity. The 100 most variable TFs across clusters were selected to generate the heatmap. **B**, **C** TFs with inferred activity score > 2 were analyzed using GSEA by using Hallmark gene signatures in (**B**) TREM1-positive myeloid cells and **C** ACTA2-positive stromal cells. Dot size indicates the gene ratio, and color represents the FDR *q* value. **D**, **E** Circle plots depicting the **D** number and **E** strength of interactions among indicated cell populations. Pink lines denote outgoing communications from TREM1-positive myeloid cells, and light purple lines denote communications from ACTA2-positive stromal cells. **F** Chord diagram depicting significant signaling pathways from TREM1-positive myeloid cells to other cell populations. **G** Heatmap depicting differences in pathway-level communication probabilities between TREM1-positive and ACTA2-positive stromal cells versus ACTA2-negative stromal cells. **H** Circle plot depicting the cell–cell communication network mediated by SPP1 signaling pathways. **I** Histogram depicting the relative contribution of ligand–receptor pairs to the SPP1 signaling pathway. **J** Western blot analysis of M2-polarized macrophages derived from human leukemia monocytic THP-1 cells. M2 macrophages were induced using IL-4 and IL-13 for 24 h, resulting in increased expression of TREM1, DAP12, and SPP1 compared with baseline controls. **K** Pharmacological inhibition of TREM1 signaling was performed using VDTJ for 24 h, followed by assessment of protein expression. Western blot results show protein levels of TREM1, DAP12, and SPP1 in control versus VDTJ-treated THP-1 cells, demonstrating that TREM1 inhibition suppresses downstream SPP1 expression. **L** Chord diagram depicting significant signaling pathways from ACTA2-positive stromal cells to other cell populations. **M** Heatmap depicting differences in pathway-level communication probabilities between ACTA2-positive stromal cells interacting with TREM1-positive versus TREM1-negative myeloid cells. *TREM1* triggering receptor expressed on myeloid cells 1, *ACTA2* α-smooth muscle actin, *CRC* colorectal cancer, *CAF* cancer-associated fibroblast, *scRNA-seq* single-cell RNA sequencing, *TF* transcription factor, *GSEA* gene set enrichment analysis, *FDR* false discovery rate, *SPP1* secreted phosphoprotein 1, *DAP12* DNAX activating protein of 12 kDa

To explore the molecular mechanisms underlying this interaction, we performed transcription factor regulon analysis and cell–cell communication modeling. TREM1-positive myeloid cells were enriched for NF-κB and AP-1 regulons (Fig. 6A, B), whereas ACTA2-positive stroma cells demonstrated activation of SMAD2/3, TWIST1, and SNAI2 transcriptional programs, indicating TGF-β-driven fibroblast activation (Fig. 6A, C). CellChat analysis identified SPP1 signaling as the dominant outgoing pathway from TREM1-positive myeloid cells to ACTA2-positive CAFs (Fig. 6D–H; Supplementary Table 6). Within this axis, the SPP1–CD44 ligand–receptor pair emerged as the principal contributor to immune–stromal communication (Fig. 6I). Notably, SPP1–CD44 signaling was also implicated in interactions between TREM1-positive myeloid cells and T cells (Supplementary Fig. 5A). In addition, NECTIN2–TIGIT (T-cell immunoreceptor with immunoglobulin and ITIM domain) signaling was identified as a key pathway connecting T cells with both TREM1-positive myeloid cells and ACTA2-positive stromal cells (Supplementary Fig. 5). TREM2, another member of the TREM family, has been reported to positively correlate with SPP1 expression in both malignant and non-malignant conditions [33, 34]. Consistent with these findings, our scRNA-seq dataset also demonstrated a positive correlation between TREM2 and SPP1 expression in CRC-associated myeloid cells (Supplementary Fig. 6A–D). However, in contrast to TREM1-positive cells, TREM2-positive cells were predominantly enriched in earlier-stage differentiated myeloid populations (Supplementary Fig. 6E).

**Fig. 7:**
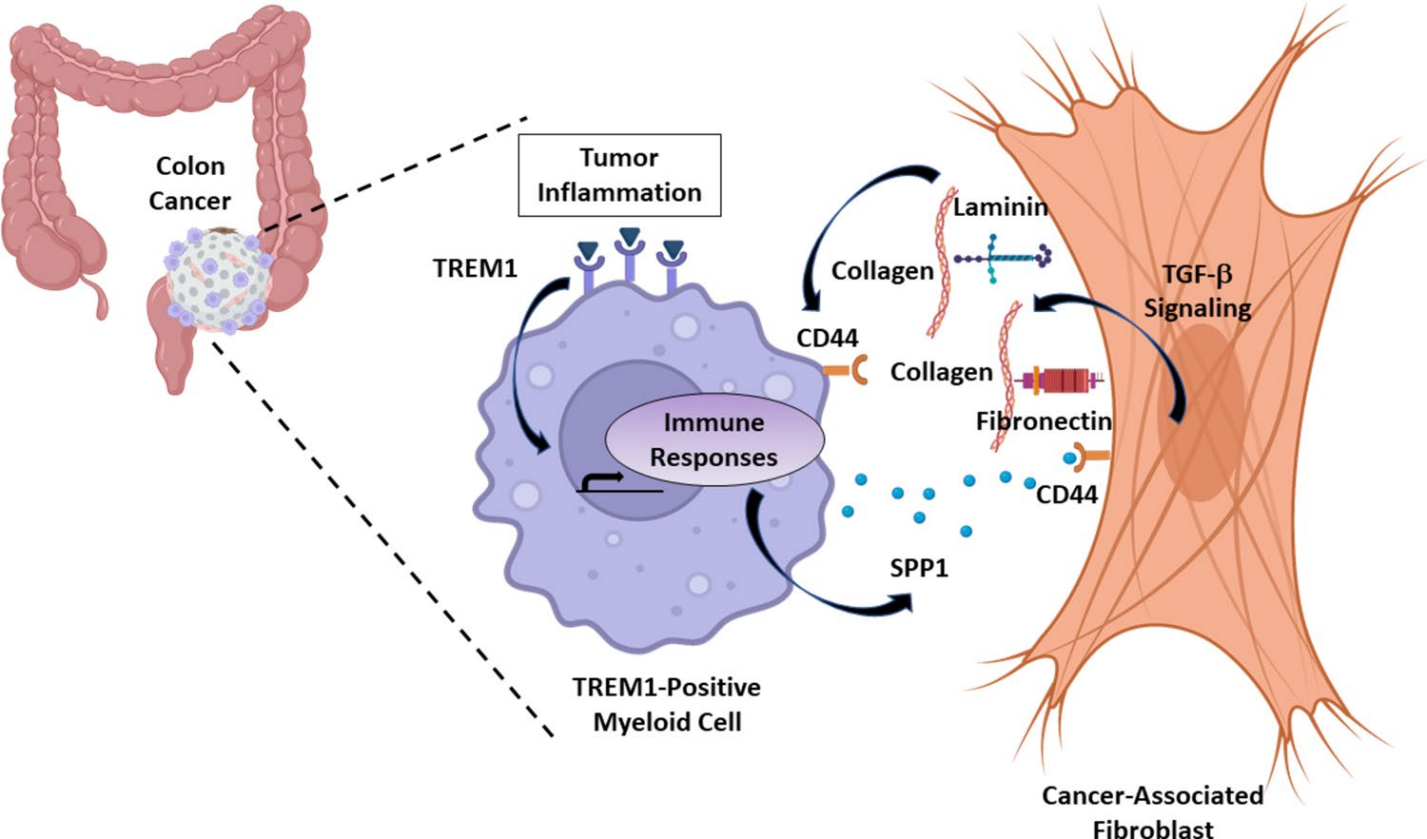
Proposed working model illustrating the crosstalk between TREM1-positive myeloid cells and CAFs in CRC. TREM1-positive TAMs activate CAFs through SPP1–CD44 signaling, whereas CAFs reinforce TAM function through ECM–CD44 interactions. This bidirectional communication establishes a profibrotic and immunosuppressive TME characteristic of MSS CRC. *TREM1* triggering receptor expressed on myeloid cells 1, *CAF* cancer-associated fibroblast, *CRC* colorectal cancer, *TAM* tumor-associated macrophage, *SPP1* secreted phosphoprotein 1, *TGF-β *transforming growth factor beta, *ECM* extracellular matrix, *TME* tumor microenvironment, *MSS* microsatellite stable

Functional validation supported these computational predictions. Our in vitro studies demonstrated that M2 polarization of THP-1 macrophages led to marked upregulation of TREM1, its adaptor protein DAP12, and SPP1 (Supplementary Fig. 7A; Fig. 6J). Conversely, TREM1 knockdown significantly reduced SPP1 expression (Supplementary Fig. 7B). Pharmacological inhibition of the TREM1–DAP12 signaling cascade using VJDT [35] further suppressed SPP1 expression (Fig. 6K) and downregulated M2 macrophage-associated markers (Supplementary Fig. 7C), confirming a direct regulatory link. Notably, several CAF-associated markers, including TGF-β1, IL-10, FAP, and PDGFR-α, were decreased in CCD-18Co cells, a human colon fibroblast cell line, when cocultured with M2-like THP-1 cells with TREM1 suppression (Supplementary Fig. 7D, E). Consistent with these findings, TCGA analyses demonstrated strong positive correlations between TREM1 and SPP1 expression (Supplementary Fig. 7F), and elevated SPP1 levels were associated with significantly poorer OS (Supplementary Fig. 7G). Conversely, signaling from ACTA2-positive stroma cells toward TREM1-positive myeloid cells was enriched for ECM pathways, including collagen, laminin, and fibronectin signaling (Fig. 6L, M; Supplementary Fig. 8). These ECM components—central drivers of matrix remodeling, cellular invasion, and metastatic progression [36, 37]—were positively correlated with TREM1 and SPP1 expression in the TCGA cohort (Supplementary Fig. 9). Collectively, these findings reveal a bidirectional communication axis between TREM1-positive myeloid cells and CAFs mediated by SPP1-related signaling, which sustains a profibrotic and immunosuppressive tumor microenvironment and highlights actionable therapeutic vulnerabilities in MSS CRC (Fig. 7).

## Discussion

CRC remains a major global health challenge, and the poor response of MSS tumors to ICIs highlights the need to clarify mechanisms sustaining their immunosuppressive TME. Within this environment, TAMCs and CAFs represent key stromal populations that cooperatively promote fibrosis, immune evasion, and tumor progression. Prior studies suggest that CAFs recruit monocytes and polarize them toward M2-like states, while macrophages release mediators that further activate CAFs, creating a reciprocal circuit of immunosuppression [17–19]. However, most evidence derives from bulk transcriptomic analyses that lack the resolution to define cellular heterogeneity or directional stromal–immune communication [38, 39]. Using scRNA-seq of paired CRC tumor and adjacent mucosa, we identified a distinct subset of TREM1-positive myeloid cells enriched for M2-like programs and strongly associated with CMS4 tumors, a subtype characterized by stromal activation and poor prognosis [31, 32]. Integrative analyses incorporating spatial localization, transcriptional regulon profiling, ligand–receptor modeling, and functional validation revealed that the TREM1–SPP1 axis is a central mediator of TREM1-positive myeloid–CAF crosstalk driving a protumorigenic and fibrotic microenvironment.

TREM1 is a myeloid-specific cell surface receptor expressed primarily on neutrophils, monocytes, and tissue macrophages [20, 21]. It functions as an amplifier of inflammatory signaling and is activated by both pathogen-associated and damage-associated molecular patterns. Mechanistically, TREM1 signals through the adaptor protein DAP12, whose immunoreceptor tyrosine-based activation motif is phosphorylated by SRC family kinases, leading to recruitment of SYK and activation of NF-κB, MAPK, PI3K/AKT, and JAK/STAT pathways. Through these pathways, TREM1 enhances cytokine production, promotes inflammatory gene expression, and shapes myeloid activation states. While classically studied in infections and autoimmune diseases [20], TREM1’s role in cancer has become increasingly recognized. In hepatocellular carcinoma and glioma, TREM1-positive TAMs promote tumor-supportive inflammation and polarization toward an M2-like phenotype [40, 41]. Hypoxia-induced activation through HIF-1α further increases TREM1 expression, while TREM1-positive TAMs upregulate PD-L1 to restrict CD8⁺ T-cell activity and contribute to ICI resistance [41]. Concordant with these observations, TREM1-positive myeloid cells in our CRC cohort displayed an immunosuppressive phenotype marked by strong enrichment of NF-κB target genes, emphasizing their role as immunoregulatory nodes within the CRC TME.

CAFs play a fundamental role in constructing the desmoplastic architecture of CRC. By producing ECM components, such as collagen, laminin, elastin, fibronectin, proteoglycans, and glycoproteins, CAFs generate a stiff stromal scaffold that promotes tumor progression while restricting immune cell infiltration and impairing drug delivery [36, 37]. In our dataset, ACTA2 emerged as a robust marker of activated CAFs, and ACTA2-positive stroma cells were enriched in CMS4 tumors and associated with poor prognosis. Furthermore, hallmark pathway and regulon analyses revealed strong enrichment of TGF-β signaling within ACTA2-positive tissues, consistent with TGF-β’s established role as a master regulator of CAF activation and differentiation. One of the most compelling findings from our integrative analyses was the identification of a reciprocal interaction network connecting TREM1-positive myeloid cells and ACTA2-positive CAFs. TREM1 activation has also been linked to fibroblast activation in fibrotic diseases, reinforcing its potential role in stromal remodeling [42, 43]. In CRC tissues, we observed physical colocalization of TREM1-positive macrophages with ACTA2-positive CAFs, supported by immunofluorescence imaging. Computational ligand–receptor analyses further revealed enrichment of profibrotic ECM signaling—collagen, laminin, and fibronectin pathways—between these two populations. A central mediator of this stromal–immune crosstalk was CD44, a multifunctional cell surface receptor involved in cancer stemness, ECM adhesion, and immunomodulation [44, 45]. CD44 serves as a convergence point for multiple ECM-derived ligands, enabling it to orchestrate inflammatory and profibrotic signaling programs. Through interactions with hyaluronic acid and collagen, CD44 promotes CAF activation and restricts cytotoxic immune infiltration. Our data showed that CD44 was a key hub mediating signals from both ECM components and immune-derived ligands, positioning it as a critical regulator of TREM1–CAF crosstalk.

SPP1 emerged as an additional major mediator of these interactions. SPP1 is a multifunctional ECM glycoprotein that supports tumor growth, angiogenesis, immune evasion, and desmoplasia [22, 46]. SPP1-positive macrophages represent a protumorigenic subset across multiple cancers and are strongly associated with poor prognosis. In CRC, a recent single-cell and spatial transcriptomic study demonstrated that SPP1-expressing macrophages contribute to ECM remodeling and cooperate with stromal cells to establish a desmoplastic niche [46]. RNA velocity and regulon analyses from this study further suggested that CAFs may promote the differentiation of thrombospondin-1 (THBS1)-positive inflammatory macrophages into SPP1-positive macrophages under hypoxic conditions. Consistent with these findings, other reports have described coexpression of THBS1 and TREM1 within immunosuppressive myeloid subsets [47, 48]. Collectively, these observations suggest a potential lineage continuum in which TREM1-positive myeloid cells are linked to THBS1- and subsequently SPP1-expressing macrophage states, thereby contributing to the establishment of a fibrotic TME. In our study, TREM1-positive macrophages interacted with CAFs predominantly through the SPP1–CD44 signaling axis. Furthermore, our in vitro experiments demonstrated that TREM1 expression modulates SPP1 expression, M2 macrophage polarization, and CAF activation. Together, these data support a functional association between TREM1 signaling and SPP1-mediated stromal remodeling. Nevertheless, the precise mechanistic relationship among TREM1, THBS1, and SPP1 remains to be fully elucidated. Further studies are warranted to determine whether THBS1 acts as an intermediate regulator within the TREM1–SPP1 axis in CRC-associated myeloid cells.

The identification of this TREM1–SPP1 signaling circuit has critical translational implications. Disrupting this pathway may attenuate protumorigenic inflammation, reduce CAF-driven fibrosis, and relieve immunosuppressive barriers that limit the efficacy of ICIs in MSS CRC. Preclinical studies have demonstrated the therapeutic potential of both ligand-dependent (e.g., LR12) and ligand-independent (e.g., VJDT) TREM1 inhibitors, with the latter offering advantages by bypassing heterogeneity among TREM1 ligands [21, 35]. In parallel, therapeutic strategies targeting SPP1—including monoclonal antibodies and small-molecule inhibitors that disrupt its interaction with CD44 or integrins—are actively under development [49, 50]. Notably, our findings demonstrate that pharmacologic inhibition of TREM1 with VJDT suppresses SPP1 expression. Given the established roles of SPP1 and CD44 in promoting T-cell exhaustion and CAF activation, TREM1 inhibition may confer additional immunomodulatory benefits beyond myeloid reprogramming. Furthermore, preclinical models have reported synergistic antitumor effects when anti-TREM1 or anti-SPP1 therapies are combined with PD-1 blockade, resulting in enhanced T-cell infiltration and improved tumor control [51, 52]. Collectively, these data provide a compelling rationale for future clinical studies evaluating combined targeting of the TREM1–SPP1 axis alongside ICIs to overcome therapeutic resistance in CRC.

This study has several limitations. First, although our in vitro experiments demonstrate that TREM1 expression promotes M2 macrophage polarization and CAF activation, the precise molecular mechanisms underlying regulation of the TREM1–SPP1 axis in macrophages remain to be fully elucidated. The MAPK signaling pathway—frequently activated by KRAS and BRAF mutations in CRC—is known to contribute to an immunosuppressive tumor microenvironment by promoting immune evasion, suppressing T-cell activity, and fostering TAM polarization [53, 54]. A previous study demonstrated that TREM1 promotes gastric cancer progression via activation of the MAPK signaling pathway [55]. Consistently, our KEGG pathway analyses showed significant enrichment of MAPK signaling in both TREM1-positive myeloid cells and ACTA2-positive fibroblasts, suggesting that this pathway may contribute to the immunosuppressive CRC microenvironment. However, direct mechanistic validation of MAPK involvement in TREM1-mediated stromal–immune interactions is beyond the scope of the present study and warrants further investigation. Second, the intrinsic regulatory network linking the TREM1–SPP1 axis with T-cell function remains to be clarified. Beyond SPP1–CD44 signaling, our cell–cell communication analyses identified the NECTIN2–TIGIT pathway as a potential mediator of interactions between TREM1-positive myeloid cells or ACTA2-positive stromal cells and T cells. TIGIT is an immune checkpoint receptor frequently upregulated on T cells and NK cells in CRC, where it contributes to immune evasion, T-cell exhaustion, and adverse clinical outcomes [56, 57]. Notably, a previous study using genetically modified mouse models showed that TREM1 silencing reduced markers of T-cell exhaustion, including TIGIT expression [35]. Although anti-TIGIT therapy is currently under clinical development, the potential therapeutic benefit of combining TIGIT blockade with TREM1-targeted strategies remains speculative and requires further preclinical and clinical validation. Third, similar to TREM1, TREM2 has recently emerged as an important regulator of myeloid cell function within the TME [33, 34]. In gastric cancer, TREM2-positive TAMs have been shown to promote CAF activation through secretion of TGF-β and IL-6, thereby driving ECM remodeling and tumor invasion [34]. In our study, TREM2 expression was positively correlated with SPP1 in CRC-associated myeloid cells. However, in contrast to TREM1, TREM2 expression was predominantly observed in earlier-stage differentiated myeloid populations. The functional interplay between TREM1 and TREM2, as well as their distinct roles across myeloid cell states in CRC, remains to be clarified. Finally, our functional assays support a role for TREM1-positive TAMs in promoting fibroblast activation. Specifically, when colon fibroblasts (CCD-18Co) were cocultured with M2-like THP-1 cells in which TREM1 was suppressed, the expressions of key CAF-associated markers—including TGF-β1, IL-10, FAP, and PDGFR-α—were significantly reduced compared with controls. While this reduction supports a role for TREM1 in macrophage-mediated stromal activation, CCD-18Co cells represent a normal fibroblast model and do not fully capture the phenotypic heterogeneity and stable reprogramming of bona fide CAFs derived from tumor tissues. Therefore, our findings should be interpreted as reflecting induction of a CAF-like state rather than definitive CAF biology. Further validation using primary CAFs or in vivo models will be essential to confirm the role of TREM1-positive myeloid cells in shaping the tumor stroma during CRC progression.

In conclusion, TREM1-positive myeloid cells drive ECM remodeling and cooperate with CAFs to establish a desmoplastic and immunosuppressive TME in CRC. Mechanistically, tumor-associated inflammatory mediators induce TREM1 expression and activate proinflammatory programs in myeloid cells, which in turn modulate fibroblast activation through SPP1-related signaling. Activated CAFs reinforce ECM remodeling and immunosuppressive function of myeloid cells through TGF-β-mediated pathways. Although the precise molecular details of this crosstalk warrant further investigation, these findings advance our understanding of stromal–immune interactions in CRC and highlight the TREM1–SPP1 axis as a potential pathway of biological and clinical relevance.

## Supplementary Information

Below is the link to the electronic supplementary material.

Supplementary file1 (PDF 380 KB)

Supplementary file2 (PDF 332 KB)

Supplementary file3 (PDF 265 KB)

Supplementary file4 (PDF 356 KB)

Supplementary file5 (PDF 186 KB)

Supplementary file6 (PDF 323 KB)

Supplementary file7 (PDF 320 KB))

Supplementary file8 (PDF 467 KB)

Supplementary file9 (PDF 221 KB)

Supplementary file10 (DOCX 16 KB)

Supplementary file11 (DOCX 15 KB)

Supplementary file12 (DOCX 16 KB)

Supplementary file13 (XLSX 13 KB)

Supplementary file14 (XLSX 11 KB

Supplementary file15 (XLSX 83 KB

## Abbreviations

CRC: Colorectal cancer
ICI: Immune checkpoint inhibitor
MSS: Microsatellite stable
TME: Tumor microenvironment
TAMC: Tumor-associated myeloid cell
CAF: Cancer-associated fibroblast
ECM: Extracellular matrix
TAM: Tumor-associated macrophage
scRNA-seq: Single-cell RNA sequencing
TREM1: Triggering receptor expressed on myeloid cells 1
ACTA2: Alpha smooth muscle actin
SPP1: Secreted phosphoprotein 1
NCKUH: National Cheng Kung University Hospital
FBS: Fetal bovine serum
PC: Principal components
MS: Macrophage state
EpCAM: Epithelial cell adhesion molecule
GSEA: Gene set enrichment analysis
FDR: False discovery rate
CMS: Consensus Molecular Subtypes
TCGA: The Cancer Genome Atlas
COADREAD: Colon and Rectal Cancer
TF: Transcription factor
EMEM: Eagle’s Minimum Essential Medium
DAP12: DNAX-activating protein of 12 kDa
EMT: Epithelial–mesenchymal transition
PFI: Progression-free interval
OS: Overall survival
TIGIT: T-cell immunoreceptor with immunoglobulin and ITIM domain
THBS1: Thrombospondin-1
