# Supplementary material for: Single-cell analysis reveals crosstalk between TREM1-positive myeloid cells and cancer-associated fibroblasts in colorectal cancer progression
Source: J Gastroenterol. 2026 Apr 27;61(8):1104–22. doi: 10.1007/s00535-026-02430-4 (PMC13407760; doi:10.1007/s00535-026-02430-4)

Supplementary Figure 1: Differential KEGG signaling enrichment in TREM1-positive and TREM1-negative myeloid cells, as well as ACTA2-positive and ACTA2-negative stromal cells derived from tumor samples. The dot plot illustrates the differences in selected KEGG gene signature scores, encompassing various signaling pathways, calculated using the *UCell* algorithm. (A) presents the distinctions between TREM1-positive and TREM1-negative myeloid cells, while (B) focuses on the comparison of ACTA2-positive and ACTA2-negative stromal cells, both sourced from tumor tissues. Abbreviations: TREM1, triggering receptor expressed on myeloid cells 1; ACTA2, α-smooth muscle actin.

A

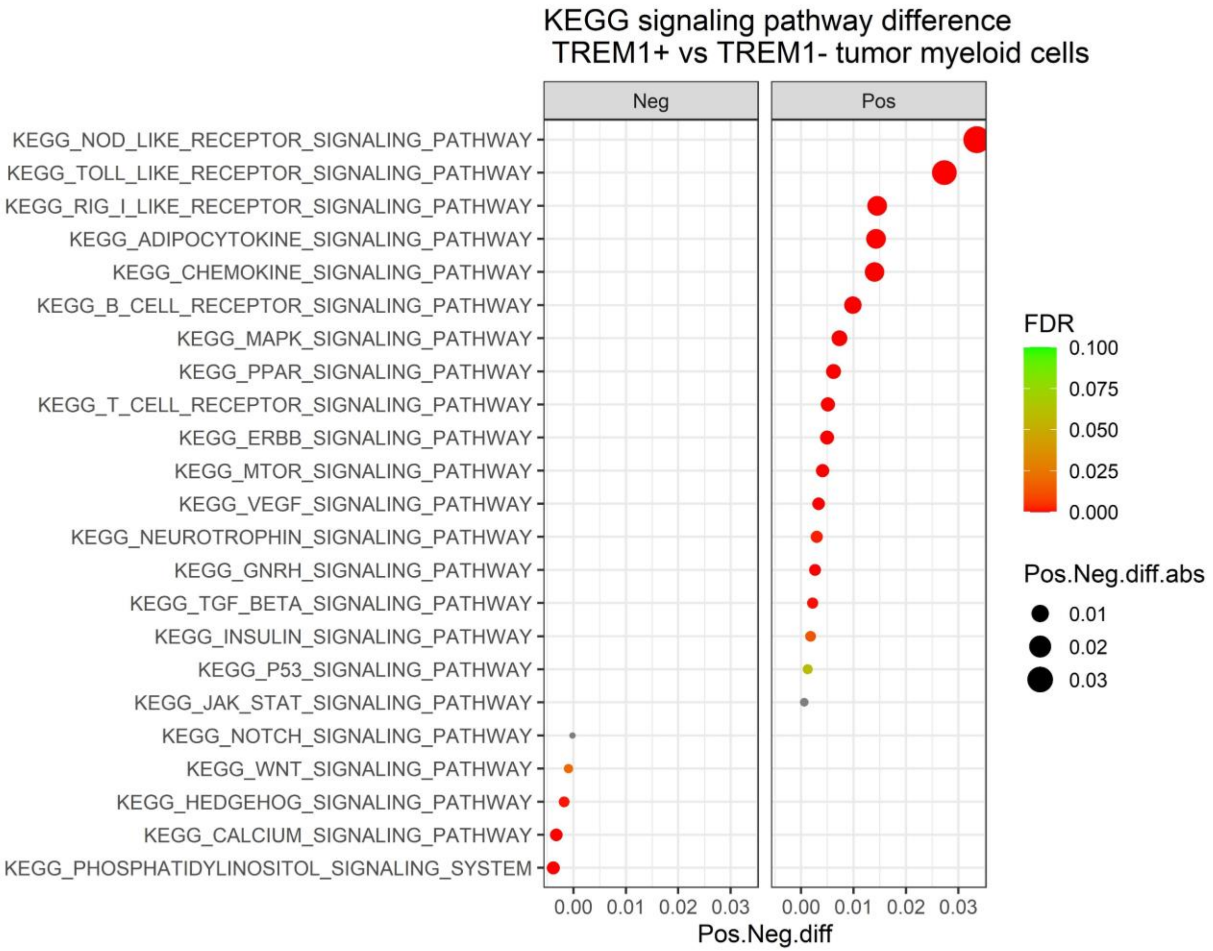

B

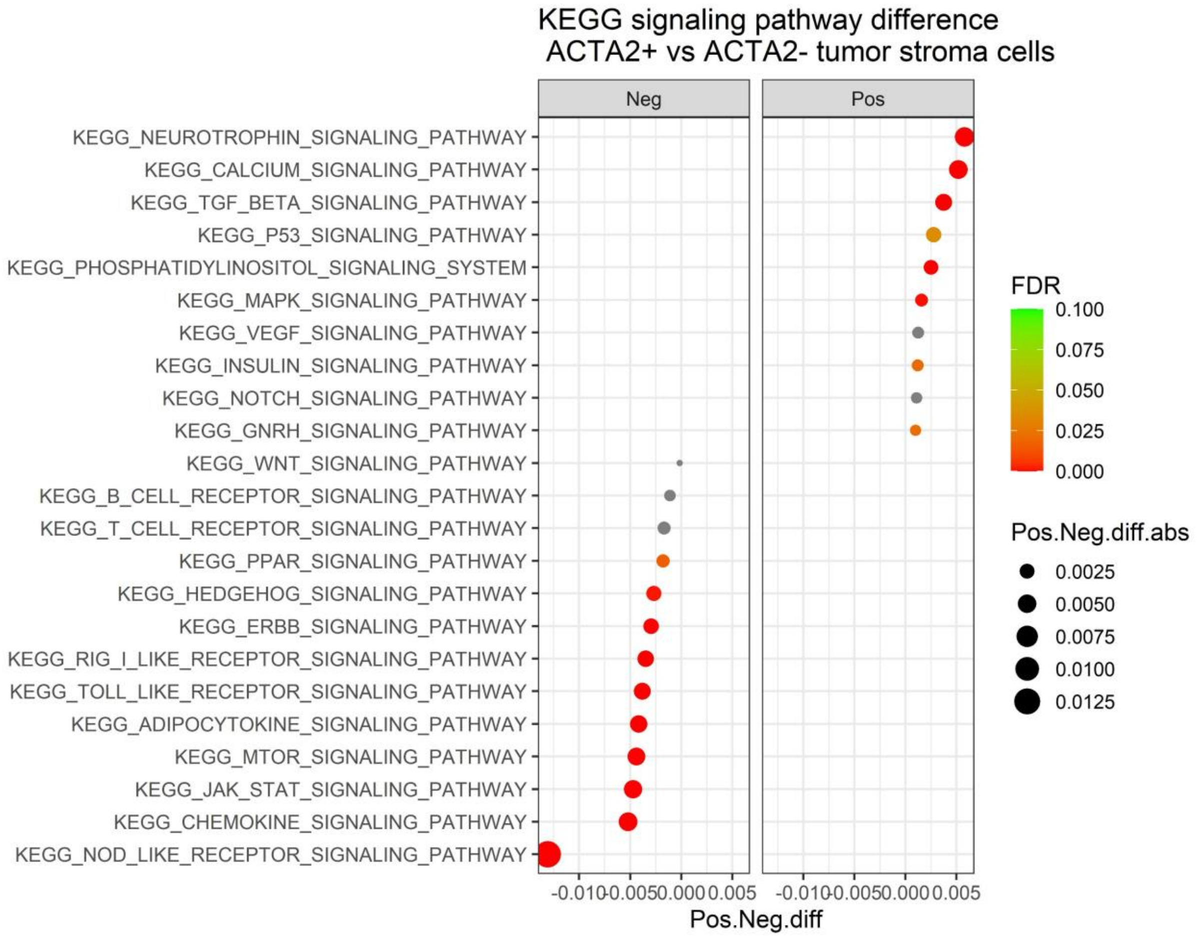

Supplement: Supplementary file 1 — Supplementary file1 (PDF 380 KB) [file 535_2026_2430_MOESM1_ESM.pdf]
