# Supplementary material for: Single-cell analysis reveals crosstalk between TREM1-positive myeloid cells and cancer-associated fibroblasts in colorectal cancer progression
Source: J Gastroenterol. 2026 Apr 27;61(8):1104–22. doi: 10.1007/s00535-026-02430-4 (PMC13407760; doi:10.1007/s00535-026-02430-4)

Supplementary Figure 2: Association of TREM1 expression with an immunosuppressive tumor microenvironment in CRC.(A) Differential expression of TREM1 between normal colorectal mucosa and tumor tissues based on TCGA transcriptomic data (UALCAN). (B–D) Scatter plots showing correlations between TREM1 expression and estimated infiltration of (B) M2 macrophages, (C) cancer-associated fibroblasts, and (D) $CD8^{+}$ T cells, as assessed using TIMER 2.0. (E) Box plots depicting TREM1 expression levels across distinct CD8+ T cell states assigned via Carcinoma EcoTyper in the TCGA COADREAD cohort. (F) Kaplan–Meier analysis illustrating the association between TREM1 expression and overall survival in patients with CRC using the KM Plotter database. Correlation coefficients and *P* values are shown in the respective panels. Abbreviations: TREM1, triggering receptor expressed on myeloid cells 1; COAD, colon adenocarcinoma; CRC, colorectal cancer; READ, rectal adenocarcinoma; TCGA, The Cancer Genome Atlas.

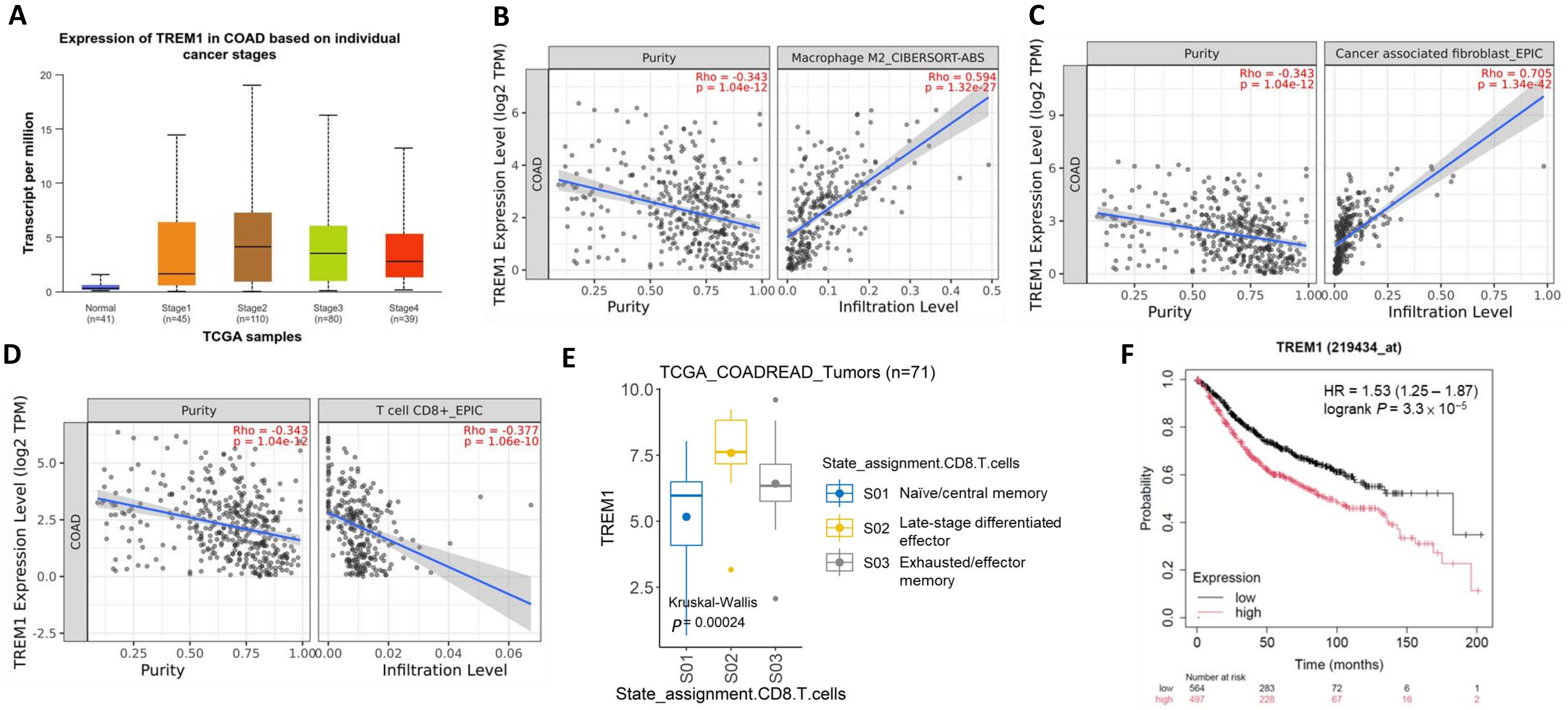

Supplement: Supplementary file 2 — Supplementary file2 (PDF 332 KB) [file 535_2026_2430_MOESM2_ESM.pdf]
