# Supplementary material for: Single-cell analysis reveals crosstalk between TREM1-positive myeloid cells and cancer-associated fibroblasts in colorectal cancer progression
Source: J Gastroenterol. 2026 Apr 27;61(8):1104–22. doi: 10.1007/s00535-026-02430-4 (PMC13407760; doi:10.1007/s00535-026-02430-4)

Supplementary Figure 3: Association of ACTA2 expression with an immunosuppressive tumor microenvironment in CRC. ACTA2 expression was analyzed using bulk transcriptomic data from TCGA. (A, B) Scatter plots showing correlations between ACTA2 expression and estimated infiltration levels of (A) CAFs and (B) M2 macrophages, as assessed by TIMER 2.0. (C) Kaplan–Meier survival analysis illustrating the association between ACTA2 expression and overall survival in CRC cohorts using the KM Plotter database. Correlation coefficients and *P* values are indicated in each panel. Abbreviations: ACTA2, α-smooth muscle actin; CRC, colorectal cancer; TCGA, The Cancer Genome Atlas; CAF, cancer-associated fibroblast.

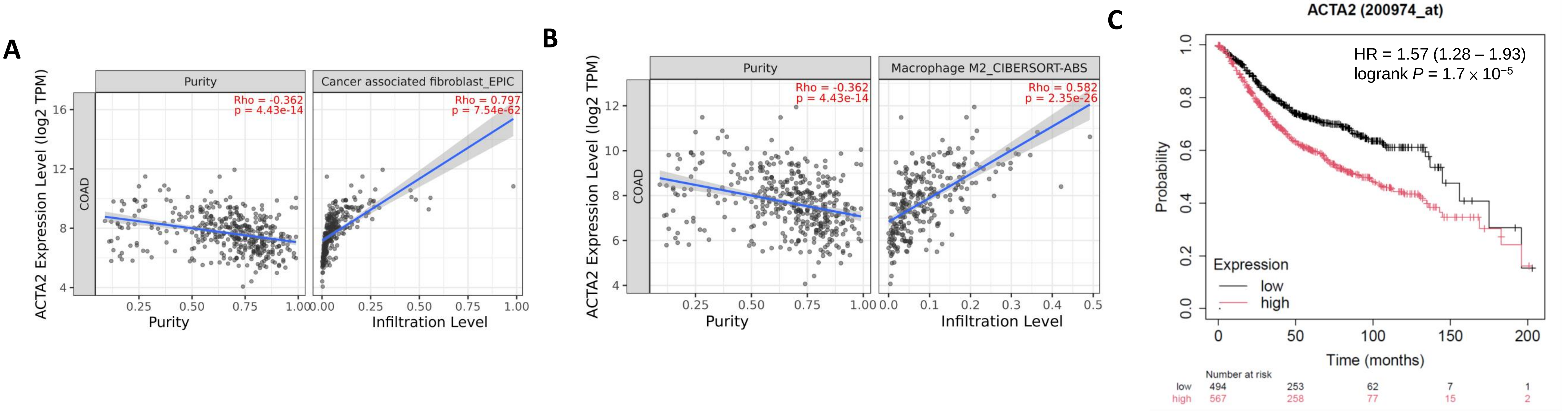

Supplement: Supplementary file 3 — Supplementary file3 (PDF 265 KB) [file 535_2026_2430_MOESM3_ESM.pdf]
