# Supplementary material for: Single-cell analysis reveals crosstalk between TREM1-positive myeloid cells and cancer-associated fibroblasts in colorectal cancer progression
Source: J Gastroenterol. 2026 Apr 27;61(8):1104–22. doi: 10.1007/s00535-026-02430-4 (PMC13407760; doi:10.1007/s00535-026-02430-4)

Supplementary Figure 4: Spatial interaction between TREM1-positive myeloid cells and ACTA2-positive stromal cells in the CRC microenvironment. Publicly available colorectal cancer Visium spatial transcriptomics data were integrated with scRNA-seq data from this study, followed by label transfer from scRNA-seq to spatial transcriptomic sections. Spatial distributions of (A) epithelial cells, (B) TREM1-positive myeloid cells, and (C) ACTA2-positive stromal cells are shown, demonstrating their spatial organization within tumor tissues. Abbreviations: TREM1, triggering receptor expressed on myeloid cells 1; ACTA2, α-smooth muscle actin; CRC, colorectal cancer; scRNA-seq, single-cell RNA sequencing.

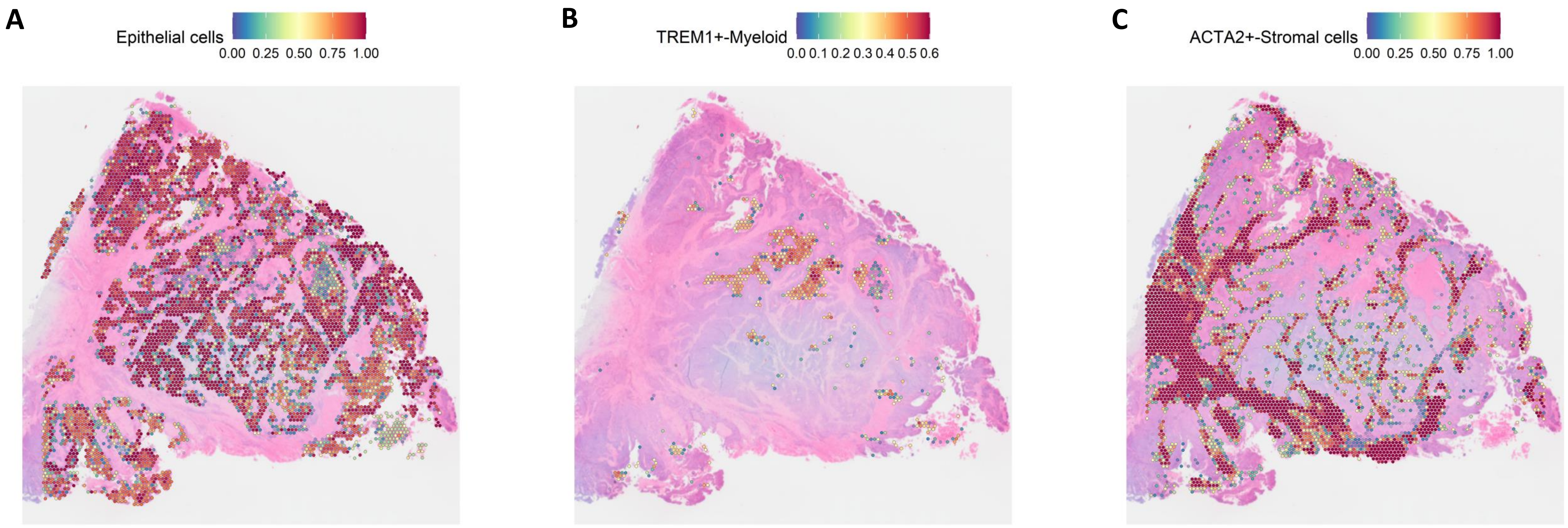

Supplement: Supplementary file 4 — Supplementary file4 (PDF 356 KB) [file 535_2026_2430_MOESM4_ESM.pdf]
