# Supplementary material for: Single-cell analysis reveals crosstalk between TREM1-positive myeloid cells and cancer-associated fibroblasts in colorectal cancer progression
Source: J Gastroenterol. 2026 Apr 27;61(8):1104–22. doi: 10.1007/s00535-026-02430-4 (PMC13407760; doi:10.1007/s00535-026-02430-4)

Supplementary Figure 5: Communication Networks from Myeloid and Stromal Cells to T Cells in the CRC TME. (A) The bubble plot illustrates significant interactions mediated by ligand-receptor pairs, including TREM1-positive and TREM1-negative myeloid cells communicating with T cells, as well as ACTA2-positive and ACTA2-negative stromal cells interacting with T cells, all derived from tumor tissues. (B) Hierarchy plot depicting the cell–cell communication network mediated by NECTIN signaling pathways. Abbreviations: CRC, colorectal cancer; TME, tumor microenvironment, TREM1, triggering receptor expressed on myeloid cells 1; ACTA2, α-smooth muscle actin.

.

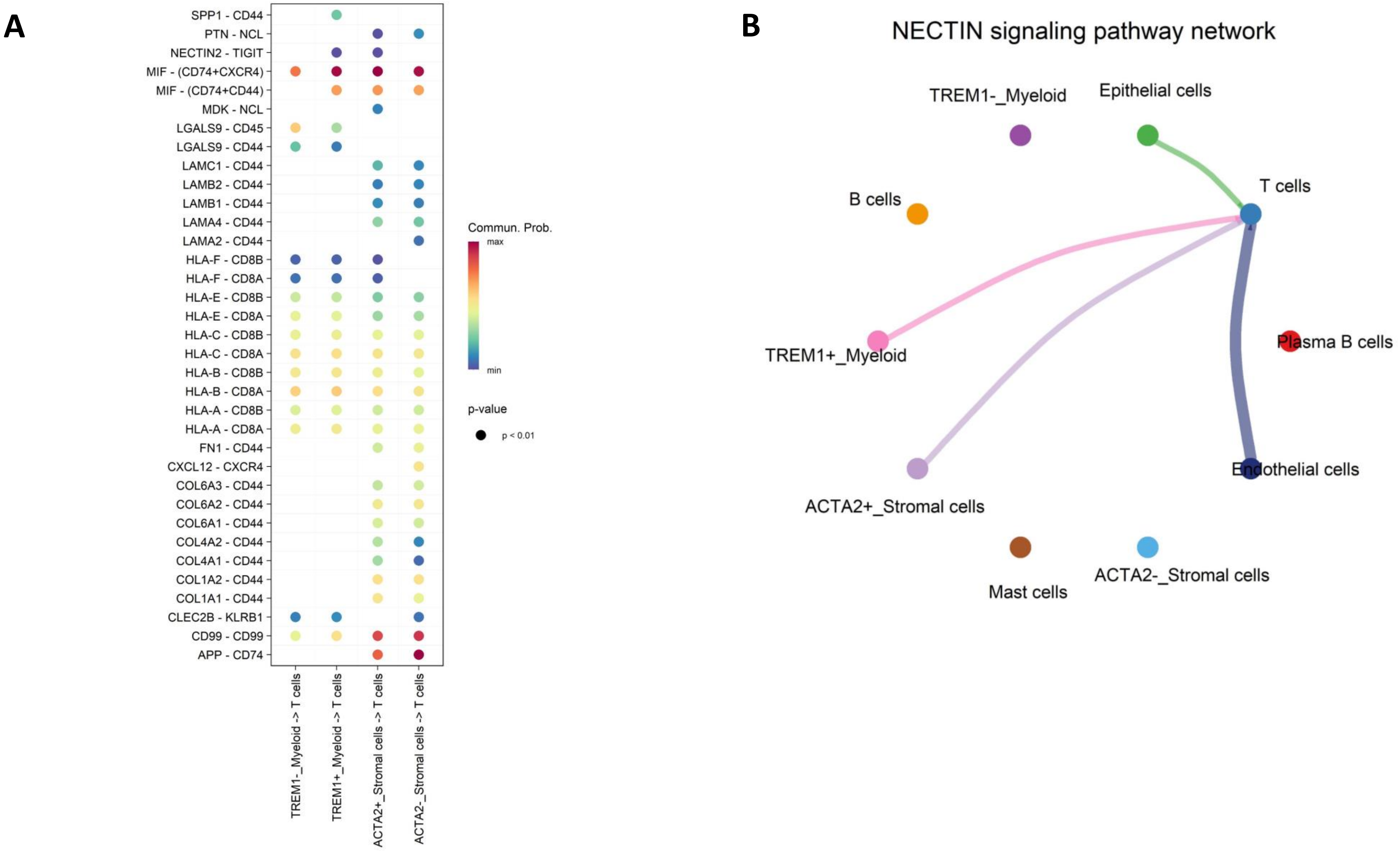

Supplement: Supplementary file 5 — Supplementary file5 (PDF 186 KB) [file 535_2026_2430_MOESM5_ESM.pdf]
