# Supplementary material for: Single-cell analysis reveals crosstalk between TREM1-positive myeloid cells and cancer-associated fibroblasts in colorectal cancer progression
Source: J Gastroenterol. 2026 Apr 27;61(8):1104–22. doi: 10.1007/s00535-026-02430-4 (PMC13407760; doi:10.1007/s00535-026-02430-4)

Supplementary Figure 6: Distribution and statistical co-occurrence of TREM1-positive, TREM2-positive, and SPP1-positive tumor myeloid cells. (A–C) UMAP projections depicting (A) SPP1-positive, (B) TREM1-positive, and (C) TREM2-positive myeloid cell populations within tumor specimens. (D) Assessment of subset overlap and concordance. The panel illustrates co-occurrence among the three myeloid signatures as determined by Fisher's exact test. (E) Bar plots illustrating the proportions of TREM1-positive (left panel) and TREM2-positive (right panel) clusters across distinct myeloid differentiation stages. Abbreviations: TREM1, triggering receptor expressed on myeloid cells 1; TREM2, triggering receptor expressed on myeloid cells 2; SPP1, secreted phosphoprotein 1; Neg, negative; Pos, positive; R, root; I, intermediate; T, terminal.

A B C

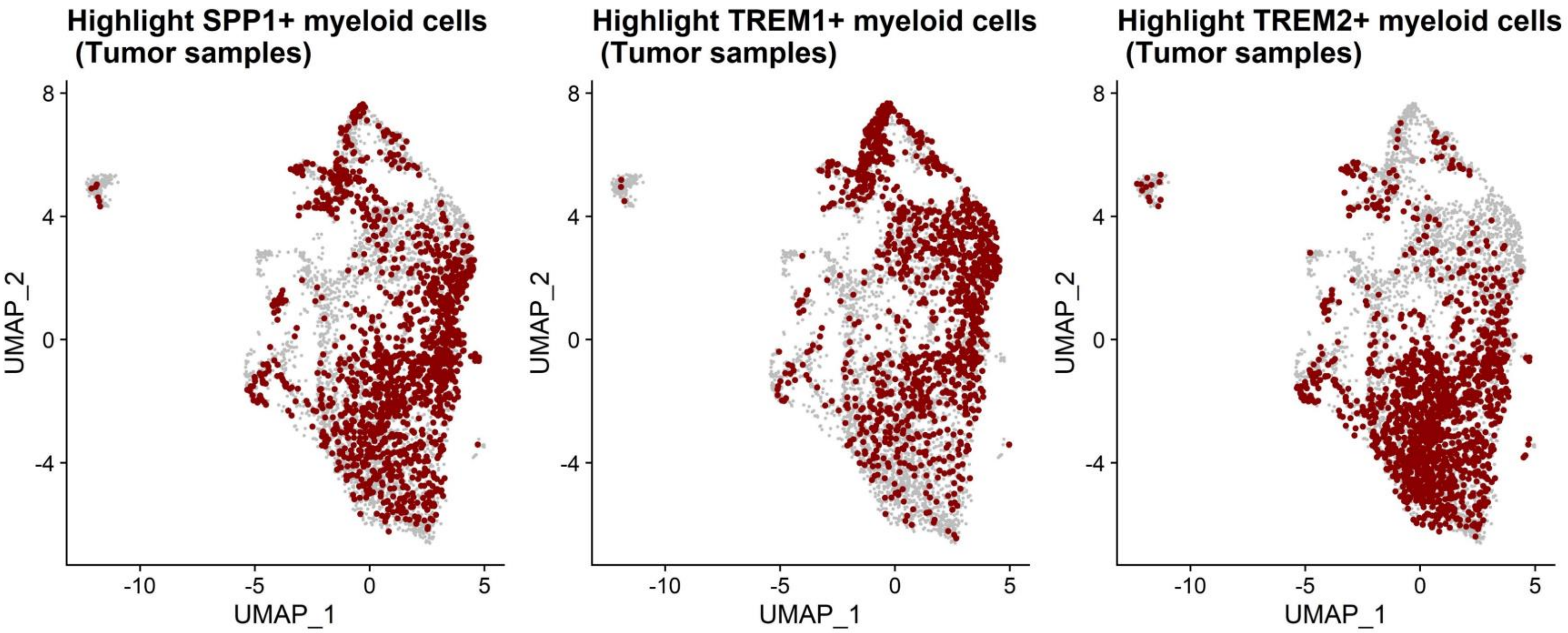

D

	Odds ratio	*P* value
TREM1 vs SPP1	3.83	$< 2 \times 10^{-16}$
TREM2 vs SPP1	4.70	$< 2 \times 10^{-16}$

E

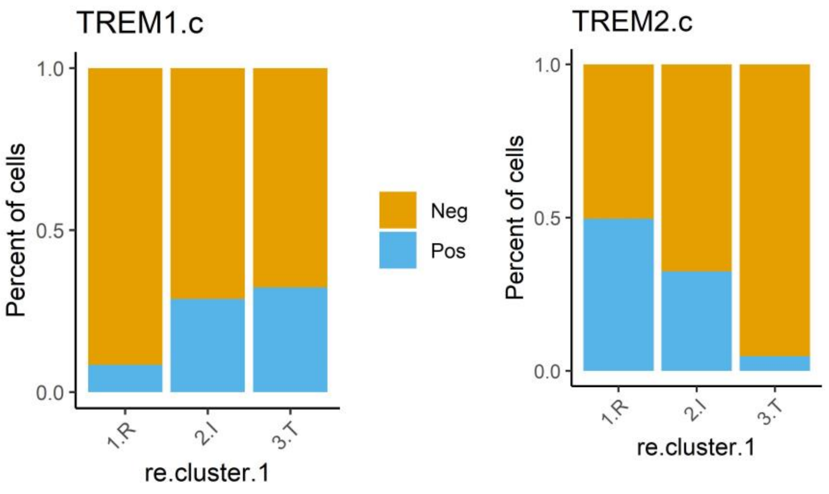

Supplement: Supplementary file 6 — Supplementary file6 (PDF 323 KB) [file 535_2026_2430_MOESM6_ESM.pdf]
