# Supplementary material for: Single-cell analysis reveals crosstalk between TREM1-positive myeloid cells and cancer-associated fibroblasts in colorectal cancer progression
Source: J Gastroenterol. 2026 Apr 27;61(8):1104–22. doi: 10.1007/s00535-026-02430-4 (PMC13407760; doi:10.1007/s00535-026-02430-4)

Supplementary Figure 7: TREM1 suppression attenuates M2 macrophage polarization and SPP1 expression**.** M2 macrophages were differentiated from THP-1 cells by stimulation with TPA, IL-4, and IL-13 for 24 h. (A) mRNA expression levels of M2 macrophage markers (IL-10, CD163, and Arg-1) were quantified by quantitative real-time PCR. (B) TREM1 was silenced using shRNA, and the effects on protein expression were assessed by Western blotting. (C) Pharmacological inhibition of TREM1 signaling was performed using VDTJ for 24 h, followed by analysis of M2 macrophage marker expression by RT-qPCR. (D, E) Expression levels of CAF-associated markers (TGFβ1, IL-10, FAP, and PDGFR-α) in CCD-18Co cells cocultured with M2-like THP-1 cells were analyzed by RT–qPCR and Western blotting. mRNA and protein were extracted from CCD-18Co cells after 24 h and 48 h of coculture, respectively. (F) Scatter plot showing a positive correlation between TREM1 and SPP1 expression in CRC tissues from TCGA. (G) Kaplan–Meier analysis demonstrating that high SPP1 expression is associated with poorer overall survival in CRC (KM Plotter). Data are presented as mean ± SD. Correlation coefficients and *P* values are shown in each panel. Abbreviations: TPA, 12-O-tetradecanoylphorbol-13-acetate; IL, interleukin; Arg-1, arginase-1; ;qPCR, quantitative polymerase chain reaction; CAF, cancer associated fibroblast; TGFβ1, transforming growth factor beta 1; FAP, fibroblast activation protein; PDGFR-α, platelet-derived growth factor receptor alpha; TREM1, triggering receptor expressed on myeloid cells 1; SPP1, secreted phosphoprotein 1; TCGA, The Cancer Genome Atlas; CRC, colorectal cancer.

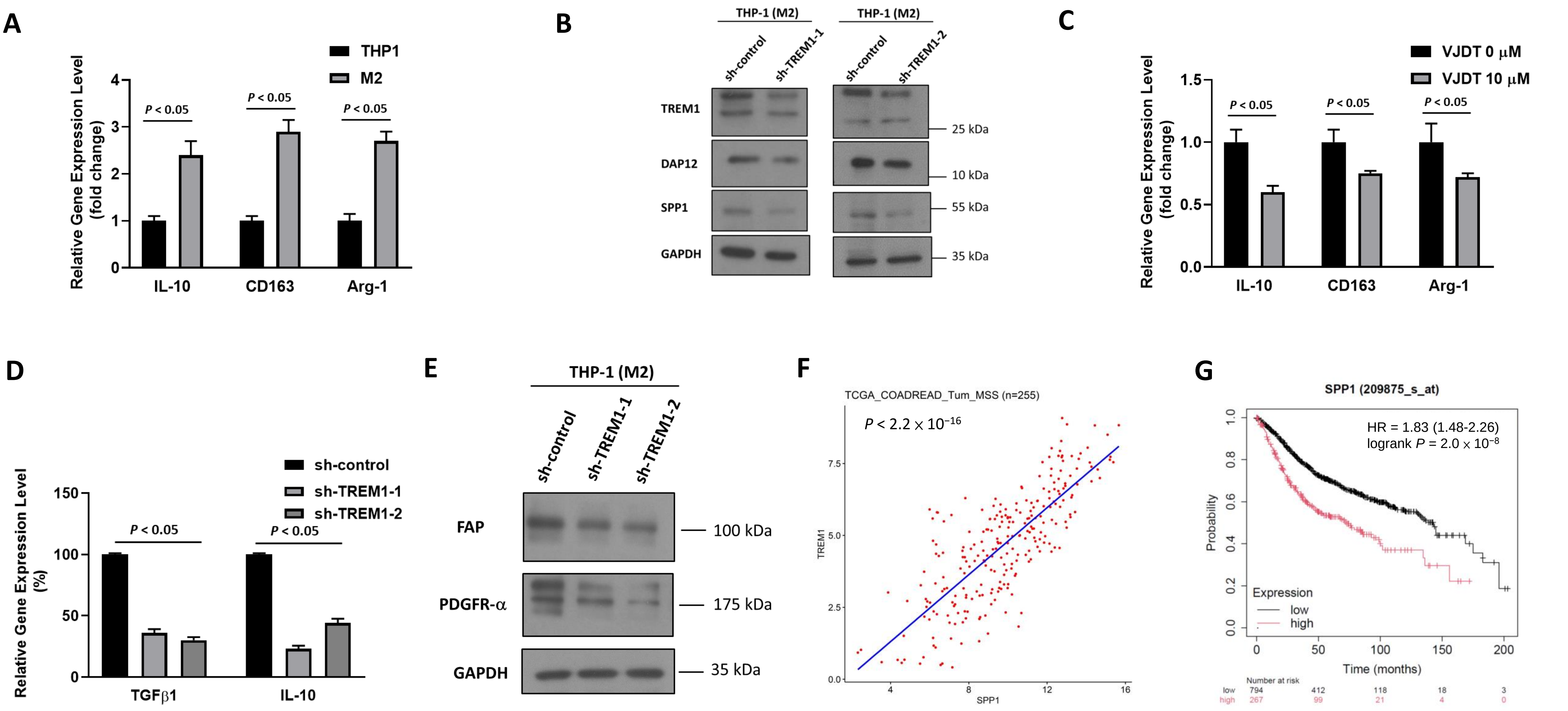

Supplement: Supplementary file 7 — Supplementary file7 (PDF 320 KB)) [file 535_2026_2430_MOESM7_ESM.pdf]
