# Supplementary material for: Single-cell analysis reveals crosstalk between TREM1-positive myeloid cells and cancer-associated fibroblasts in colorectal cancer progression
Source: J Gastroenterol. 2026 Apr 27;61(8):1104–22. doi: 10.1007/s00535-026-02430-4 (PMC13407760; doi:10.1007/s00535-026-02430-4)

Supplementary Figure 9: Positive correlations between TREM1, SPP1, and ECM-related gene expression. Scatter plots depicting Spearman correlations between gene expression levels in the TCGA datasets (n = 252), with correlation coefficients (R) and *P* values indicated. Correlations are illustrated for TREM1 with (A) COL1A2, (B) LAMA4, and (C) FN1, and for SPP1 with (D) COL1A2, (E) LAMA4, and (F) FN1. Abbreviations: TREM1, triggering receptor expressed on myeloid cells 1; SPP1, secreted phosphoprotein 1; ECM, extracellular matrix; TCGA COADREAD, The Cancer Genome Atlas colorectal adenocarcinoma; MSS, microsatellite stable; FN1, fibronectin 1.

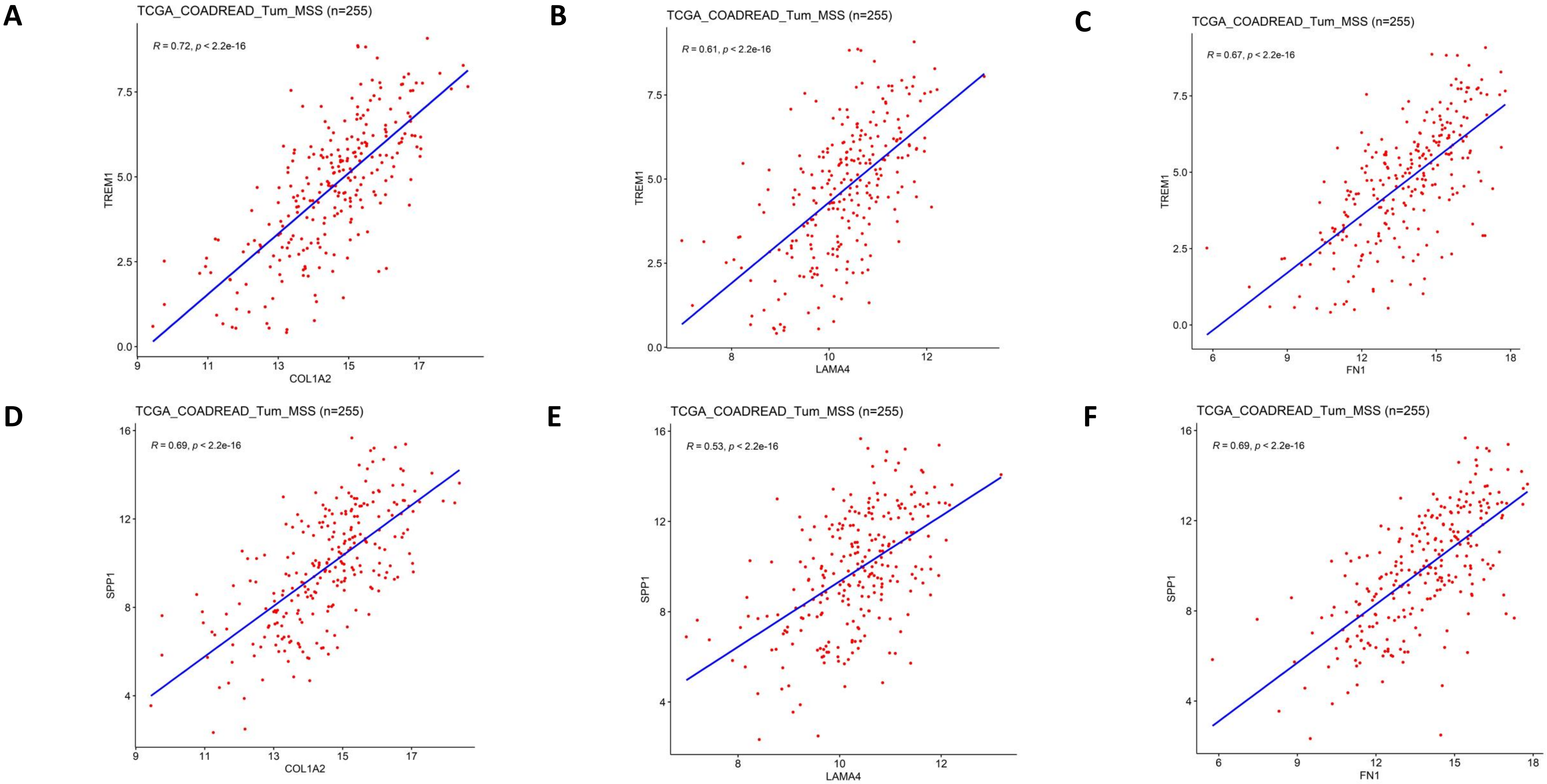

Supplement: Supplementary file 9 — Supplementary file9 (PDF 221 KB) [file 535_2026_2430_MOESM9_ESM.pdf]
