# Supplementary material for: Single-cell analysis reveals crosstalk between TREM1-positive myeloid cells and cancer-associated fibroblasts in colorectal cancer progression
Source: J Gastroenterol. 2026 Apr 27;61(8):1104–22. doi: 10.1007/s00535-026-02430-4 (PMC13407760; doi:10.1007/s00535-026-02430-4)
Supplement: Supplementary file 10 — Supplementary file10 (DOCX 16 KB) [file 535_2026_2430_MOESM10_ESM.docx]

| Antibody or reagent | Catalog number | Manufacture |
| --- | --- | --- |
| TREM1 | 11791-1-AP | Proteintech, Rosemont, Illinois, USA |
| Mix-n-Stain CF568 Antibody Labeling Kit (fluorescent dye 568) | MX568S50 | Sigma-Aldrich, St. Louis, Missouri, USA |
| ACTA2 | 53-9760-82 | ThermoFisher Scientific, Waltham, MA, USA |
| EpCAM | ab313669 | Abcam plc, Cambridge, UK |
| CD163 | 16646-1-AP | Proteintech, Rosemont, Illinois, USA |
| Mix-n-Stain CF 488A Antibody Labeling Kit (fluorescent dye 488) | MX488S50 | Sigma-Aldrich, St. Louis, Missouri, USA |
| ProLong Gold Antifade Reagent | P36935 | ThermoFisher Scientific, Waltham, MA, USA |
| 12-O-tetradecanoylphorbol-13-acetate | p8139 | Sigma-Aldrich, St. Louis, Missouri, USA |
| IL-4 | 204-IL | R&D Systems, Inc., Minneapolis, MN, USA |
| IL-13 | 213-ILB | R&D Systems, Inc., Minneapolis, MN, USA |
| VJDT | HY-157122 | MedChemExpress, Monmouth Junction, New Jersey, USA |
| DAP12 | 12492 | Cell Signaling Technology, Inc., Danvers, Massachusetts, USA |
| SPP1 | 22952-1-AP | Proteintech, Rosemont, Illinois, USA |
| FAP | 66562 | Cell Signaling Technology, Inc., Danvers, Massachusetts, USA |
| PDGFR-α | 3174 | Cell Signaling Technology, Inc., Danvers, Massachusetts, USA |
| GAPDH | 100118 | GeneTex, Inc., Irvine, CA, USA |

Supplementary Table 1. Antibody or reagent used in the experiments

Abbreviations: TREM1, triggering receptor expressed on myeloid cells 1; ACTA2, alpha smooth muscle actin; EpCAM, epithelial cell adhesion molecule; IL, interleukin; DAP12, DNAX activating protein of 12 kDa; SPP1, secreted phosphoprotein 1; FAP, fibroblast activation protein; PDGFR-a, platelet-derived growth factor receptor alpha.
