# Supplementary material for: Single-cell analysis reveals crosstalk between TREM1-positive myeloid cells and cancer-associated fibroblasts in colorectal cancer progression
Source: J Gastroenterol. 2026 Apr 27;61(8):1104–22. doi: 10.1007/s00535-026-02430-4 (PMC13407760; doi:10.1007/s00535-026-02430-4)
Supplement: Supplementary file 11 — Supplementary file11 (DOCX 15 KB) [file 535_2026_2430_MOESM11_ESM.docx]

Supplementary Table 2. Sequencing primers used in the experiments

| Gene | Primer sequence |
| --- | --- |
| IL10-F | TCAAGGCGCATGTGAACTCC |
| IL10-R | GATGTCAAACTCACTCATGGCT |
| CD163-F | TTTGTCAACTTGAGTCCCTTCAC |
| CD163-R | TCCCGCTACACTTGTTTTCAC |
| ARG1-F | TGGACAGACTAGGAATTGGCA |
| ARG1-R | CCAGTCCGTCAACATCAAAACT |
| TGFB1-F | CTAATGGTGGAAACCCACAACG |
| TGFB1-R | TATCGCCAGGAATTGTTGCTG |
| GAPDH-F | GAAGGTGAAGGTCGGAGT |
| GAPDH-R | GAAGATGGTGATGGGATTTC |
