# Supplementary material for: Single-cell analysis reveals crosstalk between TREM1-positive myeloid cells and cancer-associated fibroblasts in colorectal cancer progression
Source: J Gastroenterol. 2026 Apr 27;61(8):1104–22. doi: 10.1007/s00535-026-02430-4 (PMC13407760; doi:10.1007/s00535-026-02430-4)
Supplement: Supplementary file 12 — Supplementary file12 (DOCX 16 KB) [file 535_2026_2430_MOESM12_ESM.docx]

**Supplementary Table 3. Clinical and genomic characteristics of eight patients with colorectal cancer**

| **Patient No.** | **Age** | **Gender** | **pT** | **pN** | **pStage** | **Location** | **RAS mutation** |
| --- | --- | --- | --- | --- | --- | --- | --- |
| P02 | 57 | Male | 4b | 0 | IIC | D | WT |
| P03 | 57 | Male | 2 | 2a | IIIB | R | WT |
| P04 | 66 | Female | 3 | 0 | IIA | S | WT |
| P05 | 56 | Female | Tis | 0 | 0 | A | KRAS A146T |
| P07 | 60 | Male | 4a | 0 | IIB | A | WT |
| P08 | 76 | Female | 3 | 0 | IIA | A | WT |
| P09 | 66 | Male | 3 | 0 | IIA | S | KRAS G13D |
| P10 | 66 | Female | 3 | 0 | IIA | D | KRAS G12V |

D, descending; R, rectum; S, sigmoid; A, ascending; WT, wild type
