# Supplementary material for: Single-cell analysis reveals crosstalk between TREM1-positive myeloid cells and cancer-associated fibroblasts in colorectal cancer progression
Source: J Gastroenterol. 2026 Apr 27;61(8):1104–22. doi: 10.1007/s00535-026-02430-4 (PMC13407760; doi:10.1007/s00535-026-02430-4)

Supplementary Figure 8: Signaling pathways originating from ACTA2-positive stromal cells to distinct cellular populations. Circle plots (left) depict cell–cell communication networks mediated by the indicated signaling pathways, and histograms (right) depict the contributions of ligand–receptor pairs to (A) collagen, (B) laminin, and (C) FN1 signaling. Abbreviations: ACTA2, α-smooth muscle actin; FN1, fibronectin 1.

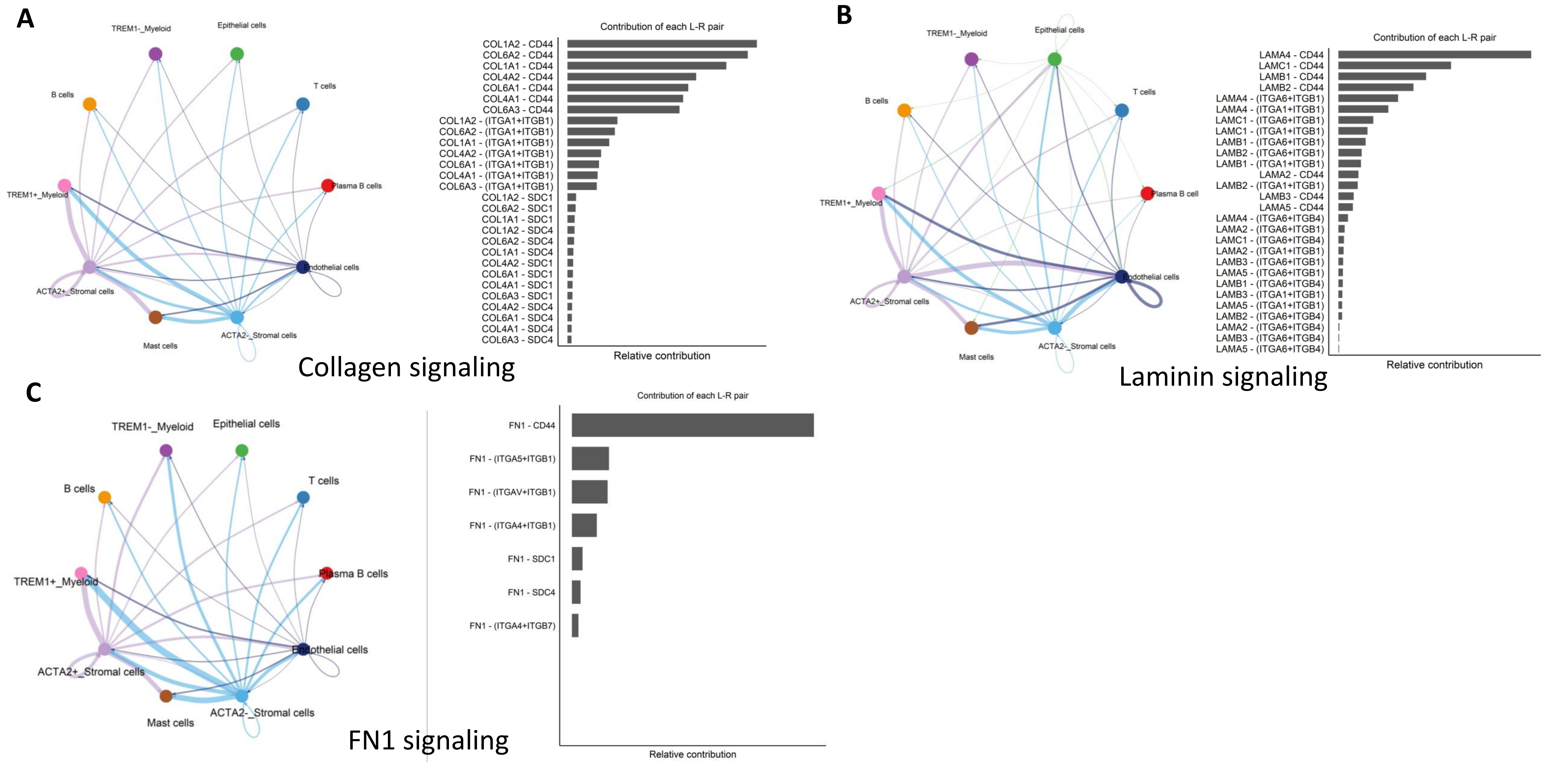

Supplement: Supplementary file 8 — Supplementary file8 (PDF 467 KB) [file 535_2026_2430_MOESM8_ESM.pdf]
